# Modeling Fibroblast–Cardiomyocyte Interactions: Unveiling the Role of Ion Currents in Action Potential Modulation

**DOI:** 10.3390/ijms252413396

**Published:** 2024-12-13

**Authors:** Yuqing Dong, Fusheng Liu

**Affiliations:** 1 The Key Laboratory of Biomedical Information Engineering of Ministry of Education, School of Life Science and Technology, Xi’an Jiaotong University, Xi’an 710049, China; dongyuqing@xjtu.edu.cn; 2 Bioinspired Engineering and Biomechanics Center, Xi’an Jiaotong University, Xi’an 710049, China; 3 State Key Laboratory for Strength and Vibration of Mechanical Structures, Xi’an Jiaotong University, Xi’an 710049, China

**Keywords:** engineered heart tissue, mathematical electrophysiological models, fibroblast–cardiomyocyte interactions, ion channels

## Abstract

Fibrotic cardiomyopathy represents a significant pathological condition characterized by the interaction between cardiomyocytes and fibroblasts in the heart, and it currently lacks an effective cure. In vitro platforms, such as engineered heart tissue (EHT) developed through the co-culturing of cardiomyocytes and fibroblasts, are under investigation to elucidate and manipulate these cellular interactions. We present the first integration of mathematical electrophysiological models that encapsulate fibroblast–cardiomyocyte interactions with experimental EHT studies to identify and modulate the ion channels governing these dynamics. Our findings resolve a long-standing debate regarding the effect of fibroblast coupling on cardiomyocyte action potential duration (APD). We demonstrate that these seemingly contradictory outcomes are contingent upon the specific properties of the cardiomyocyte to which the fibroblast is coupled, particularly the relative magnitudes of the fast Na^+^ and transient outward K^+^ currents within the cardiomyocyte. Our results emphasize the critical importance of detailed ionic current representation in cardiomyocytes for accurately predicting the interactions between cardiomyocytes and fibroblasts in EHT. Surprisingly, complex ion channel-based models of fibroblast electrophysiology did not outperform simplified resistance–capacitance models in this analysis. Collectively, our findings highlight the promising potential of synergizing in vitro and in silico approaches to identify therapeutic targets for cardiomyopathies.

## 1. Introduction

Engineered heart tissue (EHT) constructs, composed of human cardiomyocytes derived from pluripotent stem cells in conjunction with fibroblasts, hold significant potential for advancing our understanding of the functional dynamics of native cardiac and for assessing the efficacy and safety of therapeutics for cardiomyopathies [1,2]. Nevertheless, the existing evidence indicates that these constructs may not fully replicate the functional characteristics of native heart tissue, with their performance showing variability contingent upon the duration of maturation [3,4]. In light of these findings, theoretical models are being developed to quantify the functional disparities between native tissue and EHT constructs, as well as to assess temporal changes during EHT maturation, thereby evaluating the appropriateness of these constructs for pharmacological testing.

Computational models that describe the electrophysiological properties and ion handling mechanisms of cardiomyocytes and fibroblasts have effectively clarified the impact of subcellular processes on the cellular phenotypes observed experimentally, such as action potential responses and force generation [5,6,7,8,9,10,11,12]. As models representing various cell types are coupled to depict tissue-level functionality, it is imperative to ensure that these interconnected models consistently reflect the physiology of the experimental system under examination. For instance, models based on the electrophysiology of rat cardiomyocytes should not be employed to represent cardiomyocyte function in EHT constructed from human cell lines. More subtly, there has been an ongoing effort to parameterize individual components of these electrophysiological models (e.g., ion channels [13], receptors [14], transporters [15]) based on data derived from consistent cell and tissue types (e.g., ventricular [16] vs. atrial cardiomyocytes [17]), as well as from similar species. However, traditional models of cardiac electrophysiology [6,9,18] frequently depend on data from a heterogeneous array of cells, tissues, and species. This variability in cell, tissue, and species contexts employed in the development of theoretical models has been comprehensively documented by Niederer and Smith [19], who outlined the experimental and theoretical provenance of components employed in the widely utilized ten Tusscher et al. [9] and Iyer et al. [16] cardiomyocyte models. Consequently, it is crucial to assess not only the overall suitability of the model but also the appropriateness of its individual components. Human ventricular cardiomyocyte models, parameterized with ion channel and calcium handling data derived from human experimental data by Grandi et al. [20] and more recently by O’Hara et al. [21], exemplify rigorous efforts to standardize these models. Given that membrane action potential (AP) serves as a primary phenotypic indicator of cardiomyocyte functionality, it is critical to scrutinize the individual model components that influence the shape of the cardiomyocyte AP, particularly in conjunction with fibroblasts, before employing these computational models to interpret experimental results in EHT constructs and native tissue.

In pursuit of this objective, two distinct ion channel-based models have been established to investigate the electrophysiological properties and ion handling mechanisms of isolated fibroblasts [5,8]. In these studies, a specific cardiomyocyte model was coupled with varying quantities of fibroblasts, allowing for simulations of differences in the action potential shape and duration as a function of the fibroblast density. Notably, both the cardiomyocyte and fibroblast models employed in these investigations incorporate slightly different complements of membrane ion channels, which, when combined, produce markedly different outcomes, particularly concerning the time required for the membrane potential to revert to its resting state, a metric known as action potential duration (APD). Specifically, the fibroblast model devised by Sachse et al. [8] demonstrates an increase in APD with a higher number of fibroblasts coupled to the Pandit et al. [6] rat cardiomyocyte model (PCM), whereas the MacCannell et al. [5] fibroblast model (MFB) predicts a shortening of APD with an increasing number of fibroblasts coupled to the ten Tusscher et al. [9] human cardiomyocyte model (tTCM). These contrasting findings raise several critical questions: are the observed differences attributable to the specific ion channels present in each fibroblast model, the differing cardiomyocyte models that reflect variations between human and rat action potentials, or a combination of both factors?

The fundamental representation of an electrophysiologically passive fibroblast, as presented by Kohl et al. [22], can be compared to these ion channel-based fibroblast models. In the passive formulation, the fibroblast acts as a resistor–capacitor element, taking up charge from the cardiomyocyte when its membrane voltage is lower than that of the cardiomyocyte and giving up charge when the situation is reversed. A passive fibroblast coupled with mouse ventricular cardiomyocytes [23] was used to look at the effect of the gap junction conductance and relaxation time constant on the conduction velocity [24]. However, this study was performed prior to the development of the active fibroblast models investigated here. Additionally, previous work also includes the use of the MacCannell et al. fibroblast model [5] coupled with a human atrial cell model [25] to see the effect of fibroblast properties on human atrial myocyte dynamics [26]. Consequently, one objective of this study is to compare and contrast the different fibroblast models, with a particular emphasis on identifying which model or models are most appropriate for characterizing the electrophysiology of engineered heart tissues.

In this investigation, we aim to resolve the conflicting results documented in the existing literature by deconstructing the distinct functions of individual ion channels. By elucidating the ion channels that govern how fibroblasts influence cardiomyocyte APD, we identify a novel therapeutic target. We employed model fitting to characterize trends in EHT APD and subsequently predicted changes in APD following the application of a relevant ion channel inhibitor, without the need for additional fitting. These predictions were subsequently validated in EHT. It is essential to emphasize that this research specifically investigated the influence of electrical coupling between fibroblasts and cardiomyocytes on the electrophysiological characteristics of the cardiomyocytes, intentionally omitting the potential effects of endocrine factors derived from fibroblasts.

## 2. Results

### 2.1. The Active Fibroblast Models Were Dramatically Different in Their Current–Voltage Relationships

**Figure 1:**
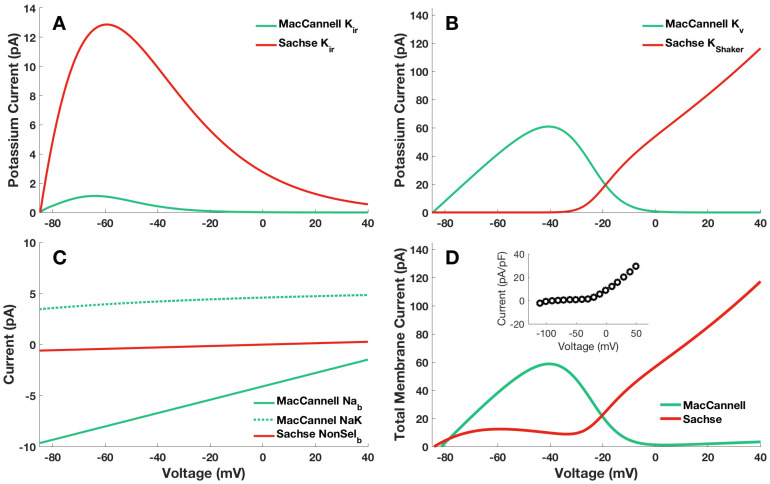
Current–voltage (I–V) relationships for individual ion channels (**A**–**C**) used in complete models (**D**) of the fibroblast developed by MacCannell et al. [5] and Sachse et al. [8]. In all subplots, the I-V relationships for the MFB model are given in green and the SFB model in blue. The inward rectifier K^+^ channel currents (*I_K_*_1_) are compared in Figure 2A. The voltage-gated K^+^ channel currents (*I_Kv_* and *I_KShkr_*) are compared in 1B. The remaining channel and exchanger currents are shown in (**C**). These are the background Na^+^ current (*I_bNa_*) and sodium–potassium exchanger current (*I_NaK_*) from MFB and the non-selective background channel current (*I_bNS_*) from SFB. The total I–V relationships for the MFB and the SFB models are shown in 2D along with experimentally obtained values of fibroblast current–voltage from Figure 2 of Shibukawa et al. [27] at [K]_o_ = 5 mM shown in the inset figure.

In Figure 1, the current–voltage relationships of each ion channel and pump used in the MFB and SFB models are shown along with the total current–voltage relationships for each fibroblast model. It is apparent that the steady-state current as a function of the voltage exhibited significant variations across these models: in the MFB, the current diminished to zero upon membrane depolarization (membrane voltage > 0), whereas the SFB demonstrated an ohmic behavior with a positive current in that voltage range. Experimental data showing the current–voltage relationship of freshly isolated left ventricular fibroblasts from Sprague-Dawley rats (inset of Figure 1, from Shibukawa et al. [27]) showed the current–voltage relationship trends of the SFB, but differed dramatically from those of the MFB model. Responses similar to those observed by Shibukawa for monocultured fibroblasts were also observed in both rat fibroblasts and myofibroblasts by Chilton et al. [28], as well as in rat neonatal fibroblasts by Rook et al. [29].

### 2.2. The tTCM/MFB Cardiomyocyte–Fibroblast Pairing Predicted Effects of Fibroblasts on Cardiomyocyte APD That Are Diametrically Opposite Those of the PCM/SFB Pairing

A comparison was performed between the two cardiomyocyte models by examining the total Na^+^, K^+,^ and Ca^2+^ currents throughout a single action potential as presented in Figure 3. The total Na^+^ current of the PCM was approximately 25% of that observed in the tTCM model; nevertheless, both models generated sufficient currents to produce a peak depolarization of around +30 mV. The total current (Figure 3A) was dominated by the fast Na^+^ current. For the total K^+^ current (Figure 3B), the PCM and tTCM models had a similar maximal peak current of 2.5 nA at the onset of depolarization; however, the decay back to a lower current (<0.20 nA) was slower with the PCM model than with the tTCM model. The transient outward K^+^ channel governed this initial spike and decay in both models. After the initial depolarization, the total K^+^ in the tTCM model was driven by the slow and rapid delayed rectifier K^+^ channels, which are replaced by the steady-state K^+^ channel in the PCM model. The total Ca^2+^ in both models was correlated to the membrane voltage so the total Ca^2+^ current in the PCM model peaked at −1.7 nA and then fell back to baseline roughly on the same time scale as the repolarization of the membrane. Conversely, the tTCM model, on the other hand, had a plateau in the total Ca^2+^ since the membrane potential remained depolarized as K^+^ slowly leaves the cell between 50 and 300 ms after depolarization. The delay in repolarization resulted in the sustained opening of the L-type Ca^2+^ channel, thereby maintaining a current during this interval.

To enable a more comprehensive analysis of the rapid sodium current (*I_Na_*) and its related charge (*q_INa_*), as well as the transient outward current (*I_to_*) and its corresponding charge (*q_Ito_*) in human and rat cardiomyocytes, represented by the tTCM and PCM models during a single action potential, the relationship between *I_Na_* and *I_to_* is illustrated in Figure 4. The simulations indicate that the peak fast sodium current *I_Na,max_* and the associated charge *q_INa_* for the tTCM model are −320 pA/pF and −0.285 pC/pF, respectively (Figure 4A), while for the PCM model, these values are −144.5 pA/pF and −0.328 pC/pF (Figure 4D). This difference is further highlighted by the peak transient outward current *I_to,max_* and its related charge *q_Ito_*, which are measured at 12.8 pA/pF and 0.287 for the tTCM model (Figure 4B) and 26.3 pA/pF and 0.894 for the PCM model (Figure 4F). Additionally, the ratio of the total charge carried by *I_to_* to that of *I_Na_* for the PCM and tTCM models is 2.72 and 1.01, respectively (Figure 4C,F). In rat cardiomyocytes, the *I_to_* current density exhibits a relatively high density, which serves to counteract the depolarizing effects of the *I_CaL_*, thereby reducing the AP plateau in these cells. The elevated ratio of peak *I_to_* to *I_Na_* appears to enhance the repolarization that occurs during phase 1 of the action potential in rat cardiomyocytes. In contrast, human cardiomyocytes demonstrate a less pronounced *I_to_* current, allowing *I_CaL_* and other potassium channels to establish a more defined plateau during phase 2 of the AP, typically following a notable depression.

Figure 5 shows the reproduced observations of MacCannell et al. [5] and Sachse et al. [8]. This set of figures showed the expected shortening of cardiomyocyte APD with the MacCannell et al. [5] coupled model and an increasing number of fibroblasts and the paradoxical lengthening of the APD in the Sachse et al. [8]. coupled model.

### 2.3. The Effect of Fibroblasts on APD Was Determined by the Cardiomyocyte Rather Than the Fibroblast

The results of maintaining the tTCM model constant while sequentially coupling it with the two different fibroblast models are illustrated in Figure 6. This was repeated with the PCM to show that the lengthening and shortening of the cardiomyocyte action potential duration when coupled with an increasing number of fibroblasts was consistently a function of the cardiomyocyte model used and appeared to be independent of the fibroblast model used. However, it can be seen that when considering the tTCM, the action potential duration was not shortened as much with an increasing number of SFBs as with the MFBs. Conversely, the PCM showed a lengthening of the APD when coupled with 1 to 10 MFBs, which was analogous to the response observed with an increasing number of SFBs. Meanwhile, we integrated the quiescent fibroblast model with both the human tTCM model and the rat PCM model, and observed comparable outcomes, as depicted in in Supplementary Materials, Figure S1.

### 2.4. A Single Cardiomyocyte Ion Channel Governed the Effects of Fibroblasts on Cardiomyocyte APD

The objective of this study was to modify the action potential characteristics of the tTCM when integrated with MFBs, in order to replicate the profile observed in the PCM and SFB coupled model. This was achieved by methodically replacing the channel formulations of the tTCM with those of the PCM, as illustrated in column 1 of Figure 7. Conversely, column 2 depicts the substitution of PCM channel formulations with tTCM channel formulations within the PCM and SFB coupled model. Although some alterations were noted in the formulations for *I_Na_*, *I_CaL_*, and *I_K_*_1_, the consistent findings included a reduction in the action potential duration in the tTCM/MFB model and an extension of the action potential duration in the PCM/SFB model, which correlated with an increasing number of parallel fibroblasts. Notably, the only instance in which these trends could be reversed occurred with the exchange of the formulation for *I_to_.*

To extend this observation further, rather than incorporating the *I_to_* conductance from the tTCM model into the PCM of the coupled PCM/SFB model, the PCM *I_to_* conductance of the PCM, denoted as *g_to_*, was systematically reduced to 50% and subsequently to 0% of its original value, as shown in Figure 8. The effect on the AP with no fibroblasts added (CM Only in Figure 8A–D) showed a marked lengthening of the action potential duration at 90% repolarization (APD_90_) from 33, to 54, 84, and then 129 milliseconds (Figure 8E), respectively. When coupled with increasing numbers of fibroblasts, there was little change in APD_90_ at a 50% blocking of *I_to_* and a shortening of APD_90_ by up to 56% of that with no coupled fibroblast at a 100% blocking of *I_to_* (Figure 8F).

### 2.5. Fibroblasts Acted as Passive Resistor–Capacitor Elements over the Timescale of Cardiomyocyte APDs

To evaluate the adequacy of a simple passive fibroblast model in relation to the AP profile of cardiomyocytes, we conducted a study involving the coupling of a single MFB with a tTCM and a single SFB with a tTCM. The simulated APs from these configurations were subsequently compared to those generated by a passive fibroblast model as described by Kohl et al. [22], which was also coupled to a tTCM. Three parameters were optimized in the passive Kohl fibroblast to best match the APs: *G_KFB_*, the nonspecific ion conductance through the membrane of the fibroblast; *V_rest_*, the resting potential of the fibroblast; and *C_m_*, the capacitance of the fibroblast. In both cases, we successfully matched the AP profile of the tTCM when coupled with either the MFB or SFB (Figure 9). The optimized values of *G_KFB_* fell within the range of previously observed values (0.1 and 4 nS), and *C_m_* supported the lower end of the range of 6.3 to 75 pF as compiled by Xie et al. [30] from previous studies. However, the predicted resting membrane potential of the fibroblast *V_rest_* was predicted to be hyperpolarized with respect to the observed isolated fibroblast *V_rest_* [31] and was found to be similar to that of the cardiomyocyte. While the action potentials of the tTCM and fibroblasts exhibited a similar shape, the time course of the gap junction current flowing between the tTCM and passive Kohl fibroblast was different in both the tTCM/MFB and tTCM/SFB cases.

### 2.6. Model Predictions Could Be Verified in Engineered Heart Tissue

To validate the simulation’s ability to mimic experimental observations on dofetilide’s inhibition of the *I_Kr_*, we compared experimental data on APD90 changes with our model’s response to *I_Kr_* blockade. The experiments involve EHT constructs, either pure cardiomyocytes or cardiomyocyte–fibroblast co-cultures, sourced from human iPSCs. We utilized both a standalone tTCM model and a coupled tTCM-SFB model. Six parameters were adjustable to match the APD_90_ experimental trends: four potassium channel conductances, one potassium pump conductance, and fibroblast gap junction resistance. The selected channels—transient outward, rapid delayed rectifier, slow delayed rectifier, and inward rectifier—were chosen for their potential reduced conductance/expression [32] in immature iPSCs, which differ from adult cardiomyocytes, the basis for the tTCM. Adjustments were consistent across both models. *I_Kr_* sensitivity to dofetilide blockade was permitted to vary due to possible fibroblast effects on dofetilide uptake. Optimal results, as shown in Figure 10, were achieved by reducing the inward rectifier conductance by 1000-fold, decreasing the slow delayed rectifier conductance by 8-fold, and doubling the rapid delayed rectifier conductance. Table 1 presents the optimized parameters alongside adult cardiac tissue values from the literature. The model accurately reflected the experimental increase in APD90 but overestimated changes in isolated cardiomyocytes and slightly underestimated those in the cardiomyocyte–fibroblast construct.

## 3. Discussion

This study clarified not only the suitability of coupled cardiomyocyte–fibroblast theoretical models for the analysis of the electrophysiological properties in EHT constructs, but also the mechanisms underlying the interactions between fibroblasts and cardiomyocytes within this framework. Three major observations were made in this regard. Firstly, the nature of the interactions between cardiomyocytes and fibroblasts were predominantly influenced by the cardiomyocytes rather than the fibroblasts themselves. The two “active” fibroblast models studied, despite their considerable differences (Figure 1), produce similar trends when coupled with the same cardiomyocyte model. These trends could be replicated with a passive representation of the fibroblast as a capacitor and resistor. Secondly, the study clarified the mechanisms through which fibroblasts impact cardiomyocytes; specifically, the alteration in the APD by fibroblasts was primarily determined by the relative magnitude of the transient outward K^+^ current in comparison to that of the fast Na^+^ current. Lastly, the coupled cardiomyocyte–fibroblast models could represent observed increases in the cardiomyocyte APD_90_ of EHT constructs exposed to dofetilide, an *I_Kr_* blocker.

We have coupled two distinct fibroblast models to two cardiomyocyte models and observed that both fibroblast models, when integrated with the same cardiomyocyte model, yield similar qualitative outcomes concerning the APD of the cardiomyocytes (Figure 6). A detailed examination of each fibroblast model revealed notable discrepancies in their current–voltage characteristics (Figure 1). This showed that the main effect of fibroblasts on the electrophysiology of cardiomyocytes is to provide a current sink or source, which is regulated by factors such as the gap junction resistance, fibroblast membrane conductance, and capacitance. Furthermore, these parameters can significantly alter the AP profile and the APD, more significantly than switching fibroblast models even though the electrophysiology in each model is distinctly different. This assertion was confirmed by replacing the MFB or SFB models with a passive resistance–capacitance model of current flow into and out of the fibroblast, as seen in Figure 9. Therefore, this simplified fibroblast model, when coupled with a cardiomyocyte model, is sufficient to describe the cardiomyocyte AP.

Regarding cardiomyocytes, it has been proposed that *I_to_* is a key determinant of APD, both in the presence and absence of fibroblast coupling [33,34,35]. This is rationalized by the fact that a substantial Ito current would precipitate repolarization during phase 1 of the cardiac action potential, thereby reducing the workload on other K^+^ channels to achieve full repolarization. However, in our coupled models, the relative balance between *I_Na_* and *I_to_*, along with the kinetics of the latter, dictates the extent of repolarization during phase 1. Moreover, the immediate decline in membrane potential [33], coupled with a high *I_to_* to *I_Na_* ratio, diminishes the influx of Ca^2+^ and abolishes the plateau phase (phase 2), a characteristic feature of the human action potential. This plateau is notably restricted in rat (and many other rodent) action potentials. These observations have been previously reviewed [24]; however, the intricate interplay between *I_to_*, *I_Na_*, and coupled fibroblasts is elucidated herein for the first time. The conductance values for *I_to_* employed in the tTCM are 45% lower than those in the PCM, aligning with experimentally determined I_to_ current densities in normal and failing human cardiomyocytes compared to normal rat cardiomyocytes [36]. However, the cell surface area of the rat cardiomyocyte in the PCM model is approximately twice that of the human cardiomyocyte in the tTCM, implying that the *I_to_* is roughly equivalent between the two models. Conversely, the *I_Na_* is significantly attenuated in the PCM model, resulting in Ito opening immediately post-depolarization without substantial *I_Na_* to counteract, thus enabling a more pronounced repolarization during phase 2. If the *I_to_* to *I_Na_* ratio were the sole determinant of the gross differences in the APD between rat and human cardiomyocyte models, then interchanging the *I_to_* formulations, which exhibit similar peak total currents, would not alter the APD as markedly as observed in Figure 7G,H. The observed discrepancy suggests that the activation and inactivation kinetics of the channels also play a pivotal role in the regulation of the APD.

Coupled cardiomyocyte–fibroblast models captured the simultaneous effects of fibroblasts and ion channel blocking agents such as the rapid delayed rectifier channel inhibitor, dofetilide. We note once again that an optimization was used to adjust the six parameters of the tTCM model. The optimization yielded conductances of the potassium channels and pumps that deviated from those of the adult tTCM human cardiomyocyte model. These specific deviations are, in retrospect, expected because the iPSC-derived cells, while cardiomyocyte-like, do vary in some important characteristics. These optimization results mirror observations from developing rat and canine cardiomyocytes as reviewed by Yang et al. [37], who noted that *I_Kr_* is the major K^+^ current that is functionally expressed in immature canine cardiomyocytes whereas *I_Ks_* seems to be absent or weakly expressed [38]. In another study, human embryonic stem cell-derived cardiomyocytes were sampled for the presence of *I_K1_* and either a small or nonexistent current density was found with a 25-fold reduction in functional expression levels [32,39]. Other observations such as the reduced functionality of calcium-induced calcium release from the sarcoplasmic reticulum [40] were not imposed on the tTCM model in this simulation. More detailed models of the electrophysiology of human iPSC-derived cardiomyocytes have been developed [41] based on experimental studies investigating the current densities and kinetics of specific ion channels [42]. These models will be important in further studies, which will try to match the automaticity of the EHT construct and the shape of the AP. Although accurate representation of the development of electrophysiological function in human iPSC-derived cardiomyocytes is important for the future revision of this system, the optimized parameters used here appear to be suitable for the present study.

We conclude with implications of the results for future efforts to discover drug targets through integrated in vitro and in silico studies. One key implication is that care must be taken in the details of the cardiomyocyte model. In the current study, the relative magnitudes of the fast Na^+^ and transient outward K^+^ currents proved to be critical, with changes to the models for the associated channels reversing the effects that fibroblasts have on cardiomyocyte APD. Clearly, efforts to make these channels match the kinetics of the native heart cardiomyocyte for which the EHT is a surrogate are important, especially when considering drugs affecting the APD. Although in this case the detailed actual current–voltage characteristics of fibroblasts were not important—fibroblasts functioned mainly as a passive resistance and capacitance element coupled to cardiomyocytes—they might be in future. Despite these challenges, the results of this study indicate that carefully integrated in silico and in vitro models can enable the identification and manipulation of new drug targets and give promise for informing future drug target searches.

## 4. Materials and Methods

### 4.1. Computational Models and Simulation Protocol

In order to address the discrepancies identified when coupling the MFB fibroblast model with the tTCM cardiomyocyte (MacCannell et al. [5]) and the SFB fibroblast model with the PCM cardiomyocyte (Sachse et al. [8]), we conducted a comparative analysis of the composition of each model. The primary distinctions between the two fibroblast models are that a Shaker-type channel is used to represent the voltage-dependent K^+^ current in the MFB model, whereas the SFB model does not incorporate a sodium–potassium pump. In addition, the formulation for *I_K1_* in the MFB is based on the cardiomyocyte *I_K1_* formulation from the tTCM [9] while in the SFB, it is based on the cardiomyocyte *I_K1_* of Iyer et al. [16]. The major currents accounted for in both cardiomyocyte models are also shown in Table 2. Both models account for calcium release and uptake by the sarcoplasmic reticulum, as well as calcium buffering within the cytosol and the sarcoplasmic reticulum. Notably, the PCM model diverges from the SFB model by substituting the slow and rapid delayed rectifier potassium currents with a steady-state potassium current (*I_Kss_*), incorporating a hyperpolarization-activated potassium–sodium current (*I_Kf_* and *I_Naf_*) and including a background potassium pump current (*I_Kb_*). These differences in the current and ion handling of the PCM are reflected in the short action potential duration observed in rat cardiomyocytes, which lack the sustained depolarized plateau characteristic of the human cardiomyocyte action potentials. In this study, four distinct cell models were integrated into four unique configurations. The units used in each model are detailed in Table S1, and conversions between different units were performed according to the specifications provided in Table S1. The computational model was simulated by MATLAB (The MathWorks Inc. (2021), Natick, MA, USA). 

All four models were reproduced to replicate the simulations given in their respective publications. The individual ion channel and pump components, as well as the total membrane current voltage relationships for each fibroblast model, were systematically compared. Additionally, the total *I_K_*, *I_Na,_* and *I_Ca_* for the cardiomyocyte models during a simulated action potential were compared between models to look for differences in the ion channel current between the adult human and rat cardiomyocytes.

**Figure 2:**
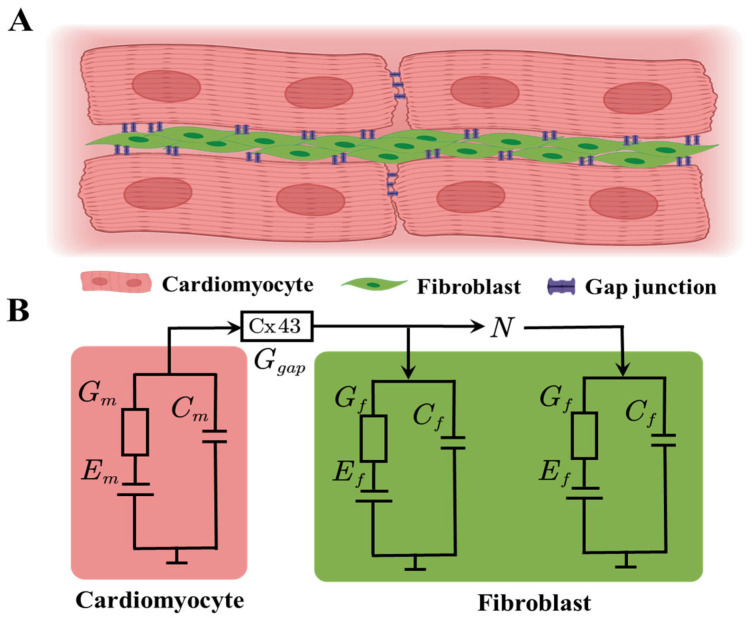
Schematic of cardiomyocyte–fibroblast coupling. A cardiomyocyte is attached to several fibroblasts along its periphery and the relative size of cardiomyocyte to fibroblast is roughly 10:1 (**A**). The electrical communication between these cells can be idealized by the cardiomyocyte in parallel with a varying number of fibroblasts (**B**). The cardiomyocyte can be represented by a variety of models (tTCM or PCM here), representing different species (human or rat, respectively), and the fibroblast can be represented by either the MacCannell et al. [5] (MFB), Sachse et al. [8] (SFB) model, or a passive resistor–capacitor element.

**Figure 3:**
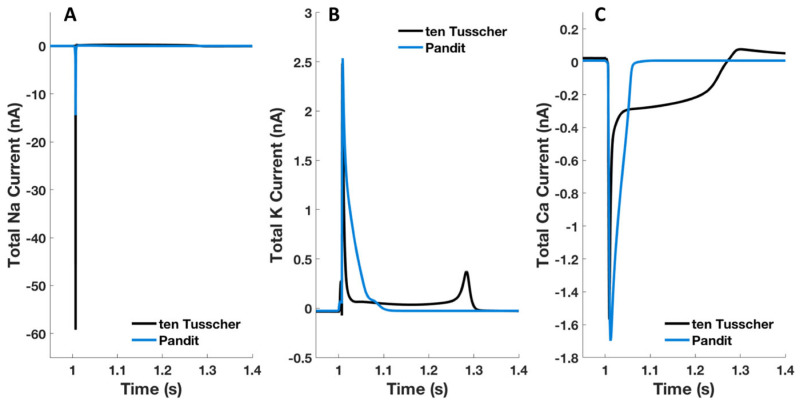
The comparison of the total Na^+^ (**A**), K^+^ (**B**), and Ca^2+^ (**C**) currents across the plasma membrane for the tTCM (black) and the PCM (blue) models. The major contributor to the total Na^+^ in both models is the fast Na^+^ current. In the case of the total K^+^ current, the transient outward K^+^ current is reflected in the initial K^+^ transient. Total Ca^2+^ current is governed by the L-type Ca^2+^ current and tracks with the membrane potential determined by Na^+^ influx and K^+^ efflux.

**Figure 4:**
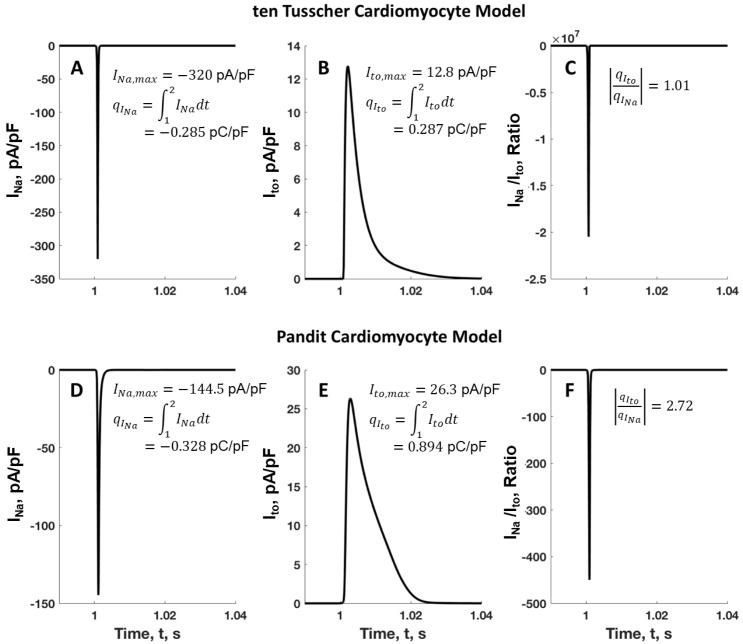
Comparison of the fast Na current *I*_Na_ and its associated charge *q_INa_*, as well as the transient outward current *I_to_* and its corresponding charge *q_Ito_* between the human and rat cardiomyocytes during a single AP lasting for a second, as modeled by the tTCM and PCM models. (**A**,**B**) represent the current through the fast Na channel and the transient outward channel for the human tTCM model, while (**D**,**E**) represent the same channels for the rat PCM model. Panels (**C**,**F**) show the ratio of *I*_Na_ to *I_to_* and the absolute value of the ratio of the total charge attributed to the transient outward and fast Na channels. Since *I*_Na_ approaches nearly zero in diastole, we chose to calculate the *I*_Na_ versus *I*_to_ ratio in this figure. Note that the currents here are scaled to the unit membrane capacitance for comparison purposes.

**Figure 5:**
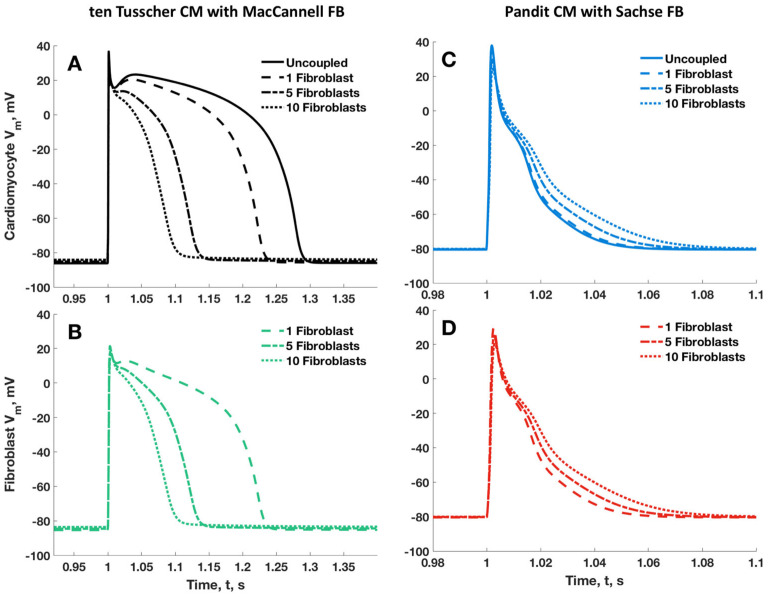
Replication of cardiomyocyte action potential and coupled fibroblast membrane potential shown in (**A**,**B**) of MacCannell et al. [5] and (**C**,**D**) of Sachse et al. [8]. Cardiomyocyte action potential is shown in (**A**,**C**) when coupled with the MFB and SFB, respectively. The associated fibroblast membrane potentials are shown in (**B**,**D**) again for the MFB and SFB, respectively. The resistances of the gap junctions between cardiomyocyte and fibroblast were set to 333 MΩ and 10 MΩ in the tTCM/MFB and PCM/SFB simulations, respectively.

**Figure 6:**
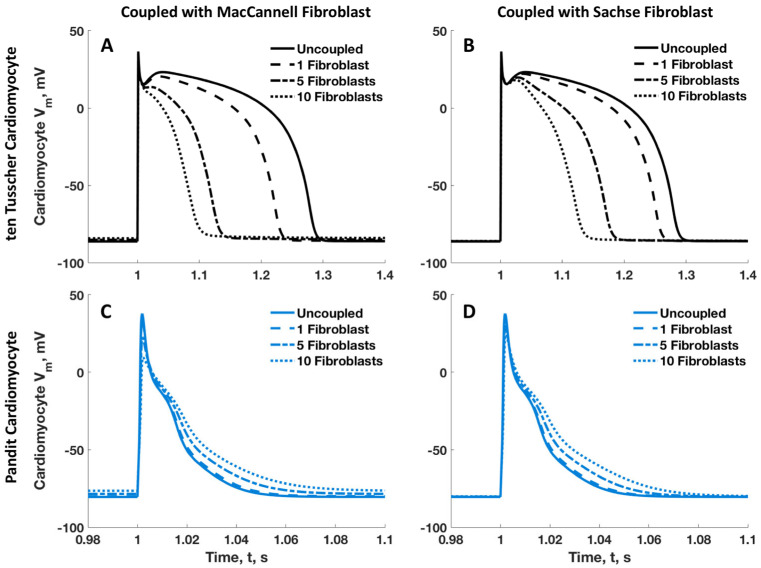
Cardiomyocyte action potential profiles when the MFB and SFB model are coupled in turn with the cardiomyocyte models of ten Tusscher et al. [9] and Pandit et al. [6]. The tTCM action potentials are shown when coupled with increasing numbers of MFBs and SFBs in (**A**,**B**), respectively. The PCM action potential when coupled similarly is shown in (**C**,**D**). Gap junction resistance between cardiomyocyte and fibroblast is held constant in all 4 simulations at 100 MΩ.

**Figure 7:**
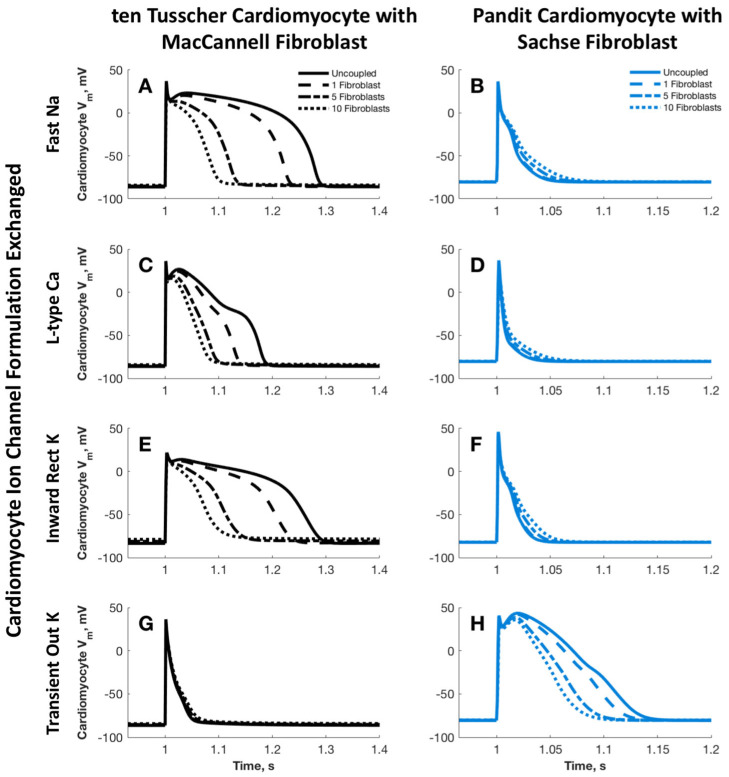
Action potential of the cardiomyocyte in the coupled model when specific ion channel current formulations are exchanged between the PCM and tTCM models. The channel current formulations that are exchanged are the fast Na^+^ (*I_Na_*), L-type Ca^2+^ (*I_CaL_*), the rapid delayed rectifier K^+^ (*I_Kr_*), and the transient outward K^+^ (*I_to_*). In column one, the tTCM is coupled with an increasing number of MFBs while in column two, the PCM is coupled to an increasing number of SFBs. The current formulations exchanged in rows one through four are *I_Na_* (**A**,**B**), *I_CaL_* (**C**,**D**), *I_Kr_* (**E**,**F**)*_,_* and *I_to_* (**G**,**H**), respectively.

**Figure 8:**
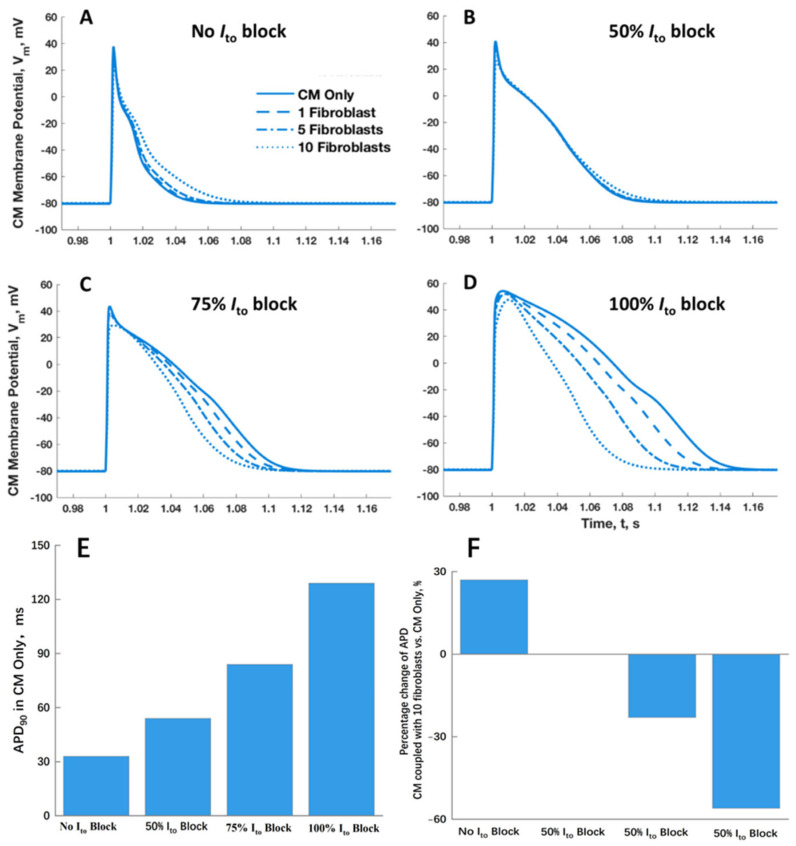
Cardiomyocyte action potential changes for the Pandit et al. [6] cardiomyocyte coupled with an increasing number of Sachse et al. [8] fibroblasts at different levels of *I_to_* conductance. For (**A**), the conductance is set at the Pandit et al. [6] *I_to_* conductance. Similarly, (**B**–**D**) shows 50%, 75%, and 0% *I_to_* conductance reflecting partial and full blockage of the transient outward K^+^ channel. (**E**) illustrates the changes in APD_90_ of cardiomyocytes without fibroblasts and (**F**) illustrates the relative change in CM when coupled with 10 fibroblasts compared to CM alone, under different levels of Ito blockade (0%, 50%, 75%, and 100%).

**Figure 9:**
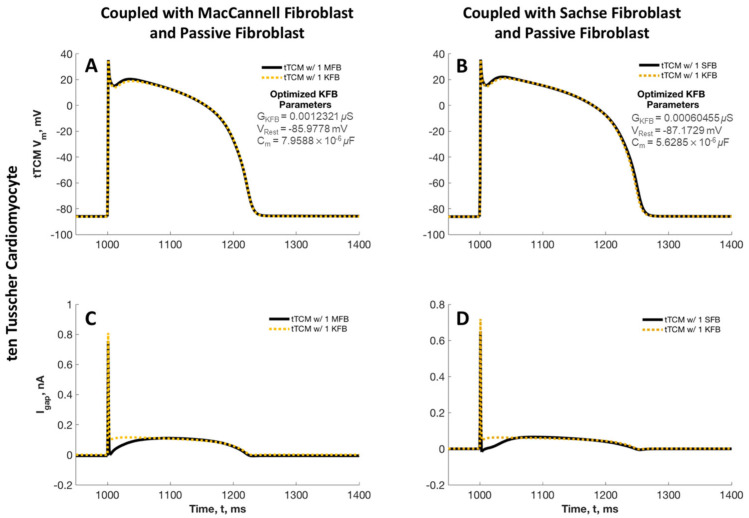
tTCM action potential and gap junction current when the tTCM is coupled with MFB, SFB, or simplified passive Kohl fibroblast (KFB) models. In (**A**), we see the tTCM AP of the tTCM/MFB model (black, solid) and the corresponding closest fit to this AP using the tTCM/KFB model with three adjustable parameters: *G_KFB_*, *V_rest,_* and *C_m_* (gold, dashed). The same is performed for the tTCM/SFB (black, solid) and best fit with the tTCM/KFB (gold, dashed) in (**B**). Gap junction currents are given in (**C**,**D**) corresponding to the results in (**A**,**B**), respectively.

**Figure 10:**
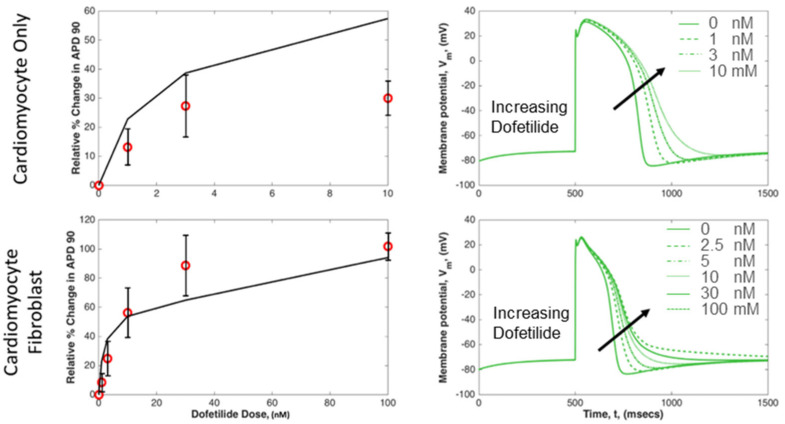
Experimental data (mean ± SEM, n = 5, red circles) and simulation results (black and green lines) of *I_Kr_* blocked with dofetilide for cardiomyocyte alone and cardiomyocyte coupled in parallel with three fibroblasts. Experimental results are for 2D constructs of human iPSC-derived cardiomyocytes alone and human iPSC-derived cardiomyocytes with fibroblasts. Simulation results are from the tTCM model for cardiomyocyte alone and the tTCM/SFB coupled model for the cardiomyocytes with fibroblasts.

In the prior investigations that integrated fibroblast and cardiomyocyte models, various configurations of connectivity between these cell types were used. However, this study specifically examines the prevalent configuration of a single cardiomyocyte coupled in parallel with an increasing number of fibroblasts, as illustrated in Figure 2. While this configuration does not account for cardiomyocyte-to-cardiomyocyte and fibroblast-to-fibroblast connections, which are significant for assessing conduction velocity, it serves as a suitable framework for investigating the impact of fibroblast quantity on the action potential of the cardiomyocyte. In the study conducted by MacCannell et al. [5], the gap junction resistance between the cardiomyocyte and fibroblast was set to 333 MΩ (conductance of 3 nS), whereas Sachse et al. [8] utilized a resistance of 100 MΩ. Both values fall within the sAme order of magnitude as intracellular resistance; Sachse et al. [8] showed that higher gap junction resistance (up to 10 GΩ) tends to insulate the two cells from each other while lower values (10 MΩ) produce similar results to those of 100 MΩ. Subsequently, the MFB and SFB were coupled with the tTCM and PCM models, respectively, resulting in a reproducible alteration in the action potential duration corresponding to the increasing number of fibroblasts, as reported in each respective study.

To investigate whether the differences in action potential duration (APD) were influenced by the fibroblast or cardiomyocyte model, we maintained a constant cardiomyocyte model while systematically substituting the two fibroblast models. In this manner, the cardiomyocyte models of the tTCM and the PCM were coupled in turn with each of the fibroblast models to establish the roles of differences in the fibroblast models. Model parameters were aligned with those specified in the original model studies (see Supplementary Material), and the gap junction resistance was standardized at 100 MΩ for each of the coupled models. It is well established that the shape of the action potential is markedly different between adult human and rat cardiomyocytes in part because the rat cardiomyocyte has a much shorter refractory period, contracting 200–300 times a minute as compared to 60 times per minute with humans.

Subsequently, we exchanged the formulations for specific ion channels among the cardiomyocyte models to investigate the impact of individual ion channels on the duration and morphology of the APD and the shape of the cardiomyocyte AP. The ion channel currents that were exchanged included fast sodium (*I_Na_*), L-type calcium (*I_CaL_*), inward rectifier potassium (*I_K_*_1_), and transient outward potassium (*I_to_*). The maximal conductance values of each of these channels in each model are shown in Table 3. Notably, the maximal conductance values for *I_CaL_* and *I_K_*_1_ in the tTCM are significantly greater by an order of magnitude than those observed in the PCM. On the other hand, the *I_Na_* maximal conductance is roughly equivalent between the two models and the *I_to_* conductance is only double the tTCM value in the PCM model. However, it is important to recognize that maximal conductance values alone do not provide a comprehensive understanding, as the fundamental formulations for the ion channels may vary between the models. For example, in the tTCM model, *I_CaL_* is formulated using the Goldman–Hodgkin–Katz equation [9], where

$$I_{CaL}=G_{CaL}dff_{Ca}\left(\frac{z^{2}VF^{2}}{RT}\right)\left(\frac{{Ca}_{i}e^{zVF/RT}-0.341{Ca}_{o}}{e^{zVF/RT}-1}\right)$$

In this expression, *I_CaL_* is the L-type Ca^2+^ current, *G_CaL_* is the channel permeability, *d*, *f,* and *f_Ca_* are the activation and inactivation gate variables, *V* is the membrane potential, *Ca_i_* is the cytosolic Ca^2+^ concentration, and *Ca_o_* is the extracellular Ca^2+^ concentration. The Ca^2+^ valence, Faraday’s constant, real gas constant, and temperature are given by *z*, *F*, *R,* and *T,* respectively. For the PCM, a simpler expression is used based on Hodgkin and Huxley [43] formalism where

$$I_{CaL}=g_{CaL}d\left[\left(0.9+\frac{{Ca}_{inact}}{10.0}\right)f_{11}+\left(0.1-\frac{{Ca}_{inact}}{10.0}\right)f_{12}\right]\left(V-E_{Ca}\right)$$

In this expression, *g_CaL_* represents the maximum conductance of the calcium channel, while the variables *d* and the expressions within brackets, including *f*_11_ and *f*_12_, define the activation and inactivation of the channel and *E_Ca_* is the fixed Nernst potential for this channel. In addition, the ion channel kinetics, as determined by the gating parameters (e.g., *d*, *f*, *f_Ca_*, *f*_11,_ and *f*_12_ in the formulations above), are different in different models; therefore, the indicator was used to look at the individual channel current or total currents for Ca^2+^ during an action potential to distinguish differences. Observations from these numerical analyses informed subsequent research aimed at modifying *I_to_* and *I_Na_*, as well as implementing *I_Kr_* blocked in coupled cardiomyocyte and fibroblast models.

### 4.2. Experimental Methods of I_Kr_ Block in EHT Constructs

To see how well the cardiomyocyte alone and the coupled cardiomyocyte–fibroblast models were able to represent experimental observation on EHT constructs, we blocked the rapid delayed rectifier channel with dofetilide and observed the changes in the action potential duration.

This experiment adhered to the Guidelines for the Care and Use of Laboratory Animals (NIH) and received approval from the Biomedical Ethics Committee at Xi’an Jiaotong University Health Science. Human cardiomyocytes were differentiated from human induced pluripotent stem cells (Applied Stem Cell, Milpitas, CA, USA). The differentiation process utilized RPMI-1640 (Gibco, Grand Island, NY, USA) supplemented with B-27 (Gibco, Grand Island, NY, USA) for the initial 7 days. Stem cell monolayers seeded in 24-well plates (Corning Inc., Corning, NY, USA) were treated with 8 µM CHIR99021 (StemRD, Burlingame, CA, USA) for a duration of 24 h, and then replaced with RPMI-1640 media. On day 3, cells were treated with 5 µM IWP-4 (Stemgent, Lexington, MA, USA) for 48 h, and then replaced with RPMI-1640 media. After day 7, the cardiomyocytes were incubated in Advanced RPMI-1640 (Gibco, Grand Island, NY, USA) to maintain contracting cardiomyocytes. Harvesting of the cardiomyocytes was performed using 0.25% Trypsin (Gibco, Grand Island, NY, USA) for 12 min, followed by aspiration and treatment with Accutase (Innovative Cell Technologies, San Diego, CA, USA) for 12 min. DMEM with 10% FBS (HyClone Laboratories, Logan, UT, USA) was added to the Accutase and the cell monolayer was resuspended by manual pipetting with a 1 mL pipette. Cardiomyocytes were filtered through a 70 µm cell strainer (Falcon/Corning Inc., Corning, NY, USA) and were centrifuged and resuspended in Advanced RPMI media and seeded in 96-well µ-plates (ibidi USA Inc., Madison, WI, USA) coated with 80 µg/mL Matrigel (Corning Inc., Corning, NY, USA). Cardiomyocytes were plated at a density of 200,000 cells per well. In wells containing both cardiomyocytes and fibroblasts, human cardiac fibroblasts (Cell Applications, Inc., San Diego, CA, USA) were introduced at a density of 200,000 cells per well within 24 h following the seeding of the cardiomyocytes. The cell cultures were sustained in Advanced RPMI 1640 for 1–2 weeks at 37 °C with 5% CO_2_ for a duration of 1–2 weeks prior to the evaluation of action potentials.

Cell cultures were incubated at 37 °C with FluVolt™ (Molecular Probes, Eugene, OR, USA) diluted to 1:500 in Advanced RPMI with 1x Powerload™ (Molecular Probes, Eugene, OR, USA). Cells were exposed to the dye at 37 °C with 5% CO_2_ for 60 min, after which the fluorescence intensity was assessed using an epifluorescence microscope to ensure the cells exhibited bright fluorescence. Subsequently, the cultures were washed twice with Advanced RPMI. The cell culture plates were incubated for an additional 15–30 min until a stable beating pattern was seen before measurements were recorded.

Action potential measurements were recorded on a high-speed kinetic plate reader FDSS/µCell (Hamamatsu, Japan) with simultaneous fluorescent detection of complete 96-well plates. The fluorescence measurements were recorded with 480 nm excitation and 540 nm emission at 100 data points/sec for 30–60 s. Normalized action potential traces recorded via fluorescence are presented in Figure S2 of the Supplementary Material.

## Figures and Tables

**Table 1:** Parameters optimized to represent *I*_Kr_ block with dofetilide and comparison to values from models and experimental observations. These six model parameters were allowed to vary from values in previous models and changes in the optimized values for K^+^ conductance reflect the trends noted in either developmental or stem cell-derived cardiomyocytes as noted in text. * Parameter values from tTCM model [9] and † from [22,31].

Adjustable Parameter Description	Symbol	Literature Value	Optimized Value	Units
Transient outward K^+^ conductance	*g_to_*	0.294 *	0.200	nS/pF
Rapid delayed rectifier K^+^ conductance	*g_Kr_*	0.096 *	0.206	nS/pF
Slow delayed rectifier K^+^ conductance	*g_Ks_*	0.245 *	0.043	nS/pF
Inward rectifier K^+^ conductance	*g_K_*_1_	5.405 *	0.006	nS/pF
Sarcolemmal K^+^ pump	*g_pK_*	0.0146 *	0.00003	nS/pF
Gap junction resistance	*R_gap_*	10–10,000 †	96.4	MΩ

**Table 2:** Complement of currents represented in each of the two fibroblast models (MacCannell et al. [5] and Sachse et al. [8]) and each of the two cardiomyocyte models (ten Tusscher et al. [9] and Pandit et al. [6]). Used currents are indicated in gray background. Channel, pumps, and currents identified in both compared model types do not mean that the current is formulated identically.

Fibroblast Models			
Description of Current	Symbol	MacCannell	Sachse
Inward rectifier K^+^	*I_K_*_1_		
Background ionic or Na^+^	*I_NSb_*, *I_Nab_*		
Voltage gated K^+^	*I_Kv_*		
Shaker K^+^	*I_KShkr_*		
Na^+^/K^+^ pump	*I_NaK_*		
**Cardiomyocyte Models**			
**Description of Current**	**Symbol**	**ten Tusscher**	**Pandit**
Fast Na^+^	*I_Na_*		
Hyperpolarization-activated Na^+^	*I_Naf_*		
L-type Ca^2+^	*I_CaL_*		
Transient outward K^+^	*I_to_*		
Slow delayed rectifier K^+^	*I_Ks_*		
Rapid delayed rectifier K^+^	*I_Kr_*		
Steady-state K^+^	*I_Kss_*		
Inward rectifier K^+^	*I_K_*_1_		
Hyperpolarization-activated K^+^	*I_Kf_*		
Na^+^/Ca^2+^ exchanger	*I_NaCa_*		
Na^+^/K^+^ pump	*I_NaK_*		
Sarcolemmal Ca^2+^ pump	*I_Cap_*		
K^+^ pump	*I_Kp_*		
Background Na^+^	*I_Nab_*		
Background K^+^	*I_Kb_*		
Background Ca^2+^	*I_Cab_*		

**Table 3:** Maximal conductance and current density parameter values for the channels, pumps, and exchangers used in the models by ten Tusscher et al. [9] (tTCM) for human and Pandit et al. [6] (PCM) for rat ventricular cardiomyocytes. The values in each model were converted to units of nS to account for differences in membrane capacitance and cell size across these models. The parameter values for the pumps and exchangers were placed into units of current per unit of membrane capacitance. * Values were variable in the formulation of the model and so were estimated by simulating the model and then backing out a comparable value of the maximal conductance or current.

Channel, Pump, or Exchanger	tTCM Model		PCM Model	
	Current	Conductance (nS)	Current	Conductance (nS)
Na^+^ Ca^2+^ K^+^	*I_Na_*	973.6	*I_Na_*	800
	*I_CaL_*	305.2 *	*I_CaL_*	31
	*I_K1_*	354.7	*I_K1_*	24
	*I_to_*	19.29	*I_to_*	35
	*I_Kr_*	6.299	*I_ss_*	7
	*I_Ks_*	16.08	---	---
	---	---	*I_f_*	1.45
K^+^ and Na^+^ Background	*I_bNa_*	0.0190	*I_bNa_*	0.0802
	*I_bCa_*	0.0388	*I_bCa_*	0.0324
	---	---	*I_bK_*	0.138
Pumps and Exchangers	*I_pCa_*	0.825 pA/pF	*I_pCa_*	0.04 pA/pF
	*I_NaCa_*	1000 pA/pF	*I_NaCa_*	0.9984 × 10^−5^ nA/mM^−4^ *
	*I_NaK_*	1.362 pA/pF	*I_NaK_*	0.800 pA/pF

## Data Availability

The original contributions presented in the study are included in the article/Supplementary Materials; further inquiries can be directed to the corresponding author.

## Supplementary Materials

The following supporting information can be downloaded at: https://www.mdpi.com/article/10.3390/ijms252413396/s1.

## References

[1] Hansen A., Eder A., Bonstrup M., Flato M., Mewe M., Schaaf S., Aksehirlioglu B., Schworer A., Uebeler J., Eschenhagen T. (2010). Development of a drug screening platform based on engineered heart tissue. Circ. Res..

[2] Wakatsuki T., Fee J.A., Elson E.L. (2004). Phenotypic screening for pharmaceuticals using tissue constructs. Curr. Pharm. Biotechnol..

[3] Hirt M.N., Boeddinghaus J., Mitchell A., Schaaf S., Bornchen C., Muller C., Schulz H., Hubner N., Stenzig J., Stoehr A. (2014). Functional improvement and maturation of rat and human engineered heart tissue by chronic electrical stimulation. J. Mol. Cell. Cardio..

[4] Schaaf S., Shibamiya A., Mewe M., Eder A., Stohr A., Hirt M.N., Rau T., Zimmermann W.H., Conradi L., Eschenhagen T. (2011). Human engineered heart tissue as a versatile tool in basic research and preclinical toxicology. PLoS ONE.

[5] MacCannell K.A., Bazzazi H., Chilton L., Shibukawa Y., Clark R.B., Giles W.R. (2007). A mathematical model of electrotonic interactions between ventricular myocytes and fibroblasts. Biophys. J..

[6] Pandit S.V., Clark R.B., Giles W.R., Demir S.S. (2001). A mathematical model of action potential heterogeneity in adult rat left ventricular myocytes. Biophys. J..

[7] Rice J.J., Wang F., Bers D.M., de Tombe P.P. (2008). Approximate model of cooperative activation and crossbridge cycling in cardiac muscle using ordinary differential equations. Biophys. J..

[8] Sachse F.B., Moreno A.P., Abildskov J.A. (2008). Electrophysiological modeling of fibroblasts and their interaction with myocytes. Ann. Biomed. Eng..

[9] ten Tusscher K.H.W.J., Noble D., Noble P.J., Panfilov A.V. (2004). A model for human ventricular tissue. Am. J. Physiol. Heart Circ. Physiol..

[10] Tran K., Smith N.P., Loiselle D.S., Crampin E.J. (2010). A metabolite-sensitive, thermodynamically constrained model of cardiac cross-bridge cycling: Implications for force development during ischemia. Biophys. J..

[11] Xie Y.F., Garfinkel A., Weiss J.N., Qu Z.L. (2009). Cardiac alternans induced by fibroblast-myocyte coupling: Mechanistic insights from computational models. Am. J. Physiol. Heart Circ. Physiol..

[12] Jacquemet V., Henriquez C.S. (2008). Loading effect of fibroblast-myocyte coupling on resting potential, impulse propagation, and repolarization: Insights from a microstructure model. Am. J. Physiol. Heart Circ. Physiol..

[13] Xu H., Guo W., Nerbonne J.M. (1999). Four kinetically distinct depolarization-activated K^+^ currents in adult mouse ventricular myocytes. J. Gen. Physiol..

[14] Bueno-Orovio A., Sánchez C., Pueyo E., Rodriguez B. (2014). Na/K pump regulation of cardiac repolarization: Insights from a systems biology approach. Pflügers Arch. Eur. J. Physiol..

[15] Bourgonje V.J., Vos M.A., Ozdemir S., Doisne N., Acsai K., Varro A., Sztojkov-Ivanov A., Zupko I., Rauch E., Kattner L. (2013). Combined Na^+^/Ca^2+^ exchanger and L-type calcium channel block as a potential strategy to suppress arrhythmias and maintain ventricular function. Circ. Arrhythm. Electrophysiol..

[16] Iyer V., Mazhari R., Winslow R.L. (2004). A computational model of the human left-ventricular epicardial myocyte. Biophys. J..

[17] Grandi E., Pandit S.V., Voigt N., Workman A.J., Dobrev D., Jalife J., Bers D.M. (2011). Human atrial action potential and Ca^2+^ model sinus rhythm and chronic atrial fibrillation. Circ. Res..

[18] Luo C.H., Rudy Y. (1994). A dynamic model of the cardiac ventricular action potential. I. Simulations of ionic currents and concentration changes. Circ. Res..

[19] Niederer S.A., Fink M., Noble D., Smith N.P. (2009). A meta-analysis of cardiac electrophysiology computational models. Exp. Physiol..

[20] Grandi E., Pasqualini F.S., Bers D.M. (2010). A novel computational model of the human ventricular action potential and Ca transient. J. Mol. Cell. Cardiol..

[21] O’Hara T., Virag L., Varro A., Rudy Y. (2011). Simulation of the undiseased human cardiac ventricular action potential: Model formulation and experimental validation. PLoS Comput. Biol..

[22] Kohl P., Kamkin A.G., Kiseleva I.S., Noble D. (1994). Mechanosensitive fibroblasts in the sino-atrial node region of rat heart: Interaction with cardiomyocytes and possible role. Exp. Physiol..

[23] Bondarenko V.E., Szigeti G.P., Bett G.C.L., Kim S.J., Rasmusson R.L. (2004). Computer model of action potential of mouse ventricular myocytes. Am. J. Physiol. Heart Circ. Physiol..

[24] Jacquemet V., Henriquez C.S. (2007). Modelling cardiac fibroblasts: Interactions with myocytes and their impact on impulse propagation. Europace.

[25] Maleckar M.M., Greenstein J.L., Giles W.R., Trayanova N.A. (2009). K^+^ current changes account for the rate dependence of the action potential in the human atrial myocyte. Am. J. Physiol. Heart Circ. Physiol..

[26] Maleckar M.M., Greenstein J.L., Giles W.R., Trayanova N.A. (2009). Electrotonic coupling between human atrial myocytes and fibroblasts alters myocyte excitability and repolarization. Biophys. J..

[27] Shibukawa Y., Chilton E.L., MacCannell K.A., Clark R.B., Giles W.R. (2005). K^+^ currents activated by depolarization in cardiac fibroblasts. Biophys. J..

[28] Chilton L., Ohya S., Freed D., George E., Drobic V., Shibukawa Y., MacCannell K.A., Imaizumi Y., Clark R.B., Dixon I.M.C. (2005). K^+^ currents regulate the resting membrane potential, proliferation, and contractile responses in ventricular fibroblasts and myofibroblasts. Am. J. Physiol. Heart Circ. Physiol..

[29] Rook M.B., van Ginneken A.C., de Jonge B., el Aoumari A., Gros D., Jongsma H.J. (1992). Differences in gap junction channels between cardiac myocytes, fibroblasts, and heterologous pairs. Am. J. Physiol. Heart Circ. Physiol..

[30] Xie Y.F., Garfinkel A., Camelliti P., Kohl P., Weiss J.N., Qu Z.L. (2009). Effects of fibroblast-myocyte coupling on cardiac conduction and vulnerability to reentry: A computational study. Heart Rhythm.

[31] Kohl P., Gourdie R.G. (2014). Fibroblast-myocyte electrotonic coupling: Does it occur in native cardiac tissue?. J. Mol. Cell. Cardiol..

[32] Huo J., Kamalakar A., Yang X., Word B., Stockbridge N., Lyn-Cook B., Pang L. (2017). Evaluation of batch variations in induced pluripotent stem cell-derived human cardiomyocytes from 2 major suppliers. Toxicol. Sci..

[33] Niwa N., Nerbonne J.M. (2010). Molecular determinants of cardiac transient outward potassium current (I-to) expression and regulation. J. Mol. Cell. Cardiol..

[34] Cordeiro J.M., Nesterenko V.V., Sicouri S., Goodrow R.J., Treat J.A., Desai M., Wu Y., Doss M.X., Antzelevitch C., Di Diego J.M. (2013). Identification and characterization of a transient outward K^+^ current in human induced pluripotent stem cell-derived cardiomyocytes. J. Mol. Cell. Cardiol..

[35] Workman A.J., Marshall G.E., Rankin A.C., Smith G.L., Dempster J. (2012). Transient outward K+ current reduction prolongs action potentials and promotes afterdepolarisations: A dynamic-clamp study in human and rabbit cardiac atrial myocytes. J. Physiol. Lond..

[36] Wettwer E., Amos G., Gath J., Zerkowski H.R., Reidemeister J.C., Ravens U. (1993). Transient outward current in human and rat ventricular myocytes. Cardiovasc. Res..

[37] Yang X.L., Pabon L., Murry C.E. (2014). Engineering adolescence: Maturation of human pluripotent stem cell-derived cardiomyocytes. Circ. Res..

[38] Obreztchikova M.N., Sosunov E.A., Plotnikov A., Anyukhovsky E.P., Gainullin R.Z., Danilo P., Yeom Z.H., Robinson R.B., Rosen M.R. (2003). Developmental changes in *I_Kr_* and *I_Ks_* contribute to age-related expression of dofetilide effects on repolarization and proarrhythmia. Cardiovasc. Res..

[39] Jonsson M.K.B., Vos M.A., Mirams G.R., Duker G., Sartipy P., de Boer T.P., van Veen T.A.B. (2012). Application of human stem cell-derived cardiomyocytes in safety pharmacology requires caution beyond hERG. J. Mol. Cell. Cardio..

[40] Sartiani L., Bettiol E., Stillitano F., Mugelli A., Cerbai E., Jaconi M.E. (2007). Developmental changes in cardiomyocytes differentiated from human embryonic stem cells: A molecular and electrophysiological approach. Stem Cells.

[41] Paci M., Hyttinen J., Aalto-Setala K., Severi S. (2013). Computational models of ventricular- and atrial-like human induced pluripotent stem cell derived cardiomyocytes. Ann. Biomed. Eng..

[42] Ma J.Y., Guo L., Fiene S.J., Anson B.D., Thomson J.A., Kamp T.J., Kolaja K.L., Swanson B.J., January C.T. (2011). High purity human-induced pluripotent stem cell-derived cardiomyocytes: Electrophysiological properties of action potentials and ionic currents. Am. J. Physiol. Heart Circ. Physiol..

[43] Hodgkin A.L., Huxley A.F. (1952). A quantitative description of membrane current and its application to conduction and excitation in nerve. J. Physiol. Lond..

