# Supplementary material for: Modeling Fibroblast–Cardiomyocyte Interactions: Unveiling the Role of Ion Currents in Action Potential Modulation
Source: Int J Mol Sci. 2024 Dec 13;25(24):13396. doi: 10.3390/ijms252413396 (PMC11677627; doi:10.3390/ijms252413396)
Supplement: Supplementary file 1 [file ijms-25-13396-s001.zip › ijms-3284376-supplementary.pdf]

Supporting information

Modeling Fibroblast-Cardiomyocyte Interactions: Unveiling the Role of Ion Currents in Action Potential Modulation

Yuqing Dong [1,2] and Fusheng Liu [2,3,*]

[1] The Key Laboratory of Biomedical Information Engineering of Ministry of Education, School of Life Science and Technology, Xi'an Jiaotong University, Xi'an 710049, China; dongyuqing@xjtu.edu.cn

[2] Bioinspired Engineering and Biomechanics Center, Xi'an Jiaotong University, Xi'an 710049, China

[3] State Key Laboratory for Strength and Vibration of Mechanical Structures, Xi'an Jiaotong University, Xi'an 710049, China

* Correspondence: sunf@greatgz.cn or liufusheng@foxmail.com

1. COMPLETE SET OF INTEGRATED MODEL EQUATIONS

In this study, four distinct cell models were integrated in four unique configurations. The units utilized in each model are detailed in Table S1, and conversions between various units were conducted in accordance with the specifications outlined in Table S1. Throughout the study, the units for the cardiomyocyte models were preserved, as modifications to these units would impact a substantial number of equations; consequently, any necessary adjustments were made to the units of the fibroblast models. For instance, when the ten Tusscher cardiomyocyte (tTCM) model was coupled with the Sacshe fibroblast (SFB) model, the time units were retained in the tTCM while being altered from seconds to milliseconds in the SFB. Conversely, in the coupling of the Pandit cardiomyocyte (PCM) with the MacCannell fibroblast (MFB), the unit of seconds was maintained, resulting in modifications solely to the equations of the MFB. The alterations in equations are documented below, while changes in fixed parameter values are presented in Section 2: Tables of Fixed Parameters. When integrating the cardiomyocyte and fibroblast models, considerations regarding intercellular current and membrane potential necessitated different approaches to the units of time and membrane capacitance for each configuration (tTCM/MFB, tTCM/SFB, PCM/SFB, and PCM/MFB). The distinct sets of expressions for each coupled model are provided at the conclusion of this section on integrated model equations.

	tTCM		PCM		SFB			MFB		
	Original	Coupled	Original	Coupled	Original	Coupled with tTCM	Coupled with PCM	Original	Coupled with tTCM	Coupled with PCM
Concentration	mM	mM	mM	mM	mM	mM	mM	mM	mM	mM
Time	ms	ms	s	s	s	ms	s	ms	ms	s
Membrane potential	mV	mV	mV	mV	mV	mV	mV	mV	mV	mV
Current	pA/pF	pA/pF	nA	nA	nA	nA	nA	pA/pF	pA/pF	pA/pF

Table S1. Units used in original model formulations by ten Tusscher et al. (tTCM), Pandit et al. (PCM), Sachse et al. (SFB) and MacCannell et al. (MFB) and the coupled model formulations presented in this study. mM = millimolar, ms = milliseconds, s = seconds, mV = millivolts, pA/pF = picoamperes per picofarad and nA = nanoamperes

Model 1: ten Tusscher et al. human cardiomyocyte model

The following set of equations delineates the functions of membrane ion channels, pumps, and transporters, as well as the role of the sarcoplasmic reticulum in calcium buffering, within the framework of human cardiomyocyte electrophysiology and ion handling, as initially described by ten Tusscher et al[1]. It is important to highlight two key points: first, the calcium buffering expressions in the cytosol and sarcoplasmic reticulum (S77 and S78) differ from those presented in the original publication and are based on the curated model available from the Physiome Model Repository (PMR); second, the equations governing the dynamics of calcium, sodium, and potassium (S79, S81, and S82) incorporate the membrane capacitance, C_m, for all currents traversing the plasma membrane.

Reversal Potentials

$$E_{Na} = \frac{RT}{F}\log\frac{Na_o}{Na_i} \tag{S1}$$

$$E_{Ca} = \frac{RT}{2F}\log\frac{Ca_o}{Ca_i} \tag{S2}$$

$$E_{K} = \frac{RT}{F}\log\frac{K_o}{K} \tag{S3}$$

$$E_{Ks} = \frac{RT}{F}\log\frac{K_o + (pKNa \cdot Na_o)}{K_i + (pKNa \cdot Na_i)} \tag{S4}$$

Fast Sodium Current

$$i_{Na} = g_{Na} \cdot m^3 \cdot h \cdot j \cdot (V_m - E_{Na}) \tag{S5}$$

Fast sodium current activation gate

$$m_\infty = \frac{1}{[1 + e^{(-56.86 - V_m)/9.03}]^2} \tag{S6}$$

$$\alpha_m = \frac{1}{1 + e^{(-60 - V_m)/5}} \tag{S7}$$

$$\beta_m = \frac{0.1}{1 + e^{(35 + V_m)/5}} + \frac{0.1}{1 + e^{(V_m - 50)/200}} \tag{S8}$$

$$\tau_m = \alpha_m \cdot \beta_m \tag{S9}$$

$$\frac{dm}{dt} = \frac{m_\infty - m}{\tau_m} \tag{S10}$$

Fast sodium current fast inactivation gate

$$h_\infty = \frac{1}{[1 + e^{(71.55 + V_m)/7.43}]^2} \tag{S11}$$

$$\alpha_h = \begin{cases} 0.057 \cdot e^{[-(80 + V_m)]/6.8} & V_m < -40 \text{ mV} \\ 0 & \text{otherwise} \end{cases} \tag{S12}$$

$$\beta_h = \begin{cases} 2.7 \cdot e^{0.079 \cdot V_m} + 310000 \cdot e^{0.3485 \cdot V_m} & V_m < -40 \text{ mV} \\ \dfrac{0.77}{0.13 \cdot 1 + e^{[-(V_m + 10.66)]/11.1}} & \text{otherwise} \end{cases} \tag{S13}$$

$$\tau_h = \frac{1}{\alpha_h + \beta_h} \tag{S14}$$

$$\frac{dh}{dt} = \frac{h_\infty - h}{\tau_h} \tag{S15}$$

Fast sodium current slow inactivation gate

$$j_\infty = \frac{1}{[1 + e^{(71.55 + V_m)/7.43}]^2} \tag{S16}$$

$$\alpha_j = \begin{cases} \dfrac{-2.5428 \cdot 10^4 \cdot e^{0.2444 \cdot V_m} - 6.948 \cdot 10^{-6} \cdot e^{-0.04391 \cdot V_m}}{1 + e^{0.311 \cdot (V_m + 79.23)}} \cdot (V_m + 37.78) & V_m < -40 \text{ mV} \\ 0 & \text{otherwise} \end{cases} \tag{S17}$$

$$\beta_j = \begin{cases} \dfrac{0.02424 \cdot e^{-0.01052 \cdot V_m}}{1 + e^{-0.1378 \cdot (V_m + 40.14)}} & V_m < -40 \text{ mV} \\ \dfrac{0.6 \cdot e^{0.057 \cdot V_m}}{1 + e^{-0.1 \cdot (V_m + 32)}} & \text{otherwise} \end{cases} \tag{S18}$$

$$\tau_j = \frac{1}{\alpha_j + \beta_j} \tag{S19}$$

$$\frac{dj}{dt} = \frac{j_\infty - j}{\tau_j} \tag{S20}$$

L-type Calcium Current

$$i_{CaL} = g_{CaL} \cdot d \cdot f \cdot f_{Ca} \cdot \frac{4V_m F}{RT} \cdot \frac{\left(Ca_i \cdot e^{2V_m F/RT}\right) - (0.341 \cdot Ca_o)}{e^{2V_m F/RT} - 1} \tag{S21}$$

L-type calcium current activation gate

$$d_\infty = \frac{1}{1 + e^{(-5 - V_m)/7.5}} \tag{S22}$$

$$\alpha_d = \frac{1.4}{1 + e^{(-35 - V_m)/513}} + 0.25 \tag{S23}$$

$$\beta_d = \frac{1.4}{1 + e^{(5 + V_m)/5}} \tag{S24}$$

$$\gamma_d = \frac{1.4}{1 + e^{(50 - V_m)/20}} \tag{S25}$$

$$\tau_d = \alpha_d \cdot \beta_d + \gamma_d \tag{S26}$$

$$\frac{dd}{dt} = \frac{d_\infty - d}{\tau_d} \tag{S27}$$

L-type calcium current voltage dependent inactivation gate

$$f_\infty = \frac{1}{1 + e^{(20 - V_m)/7}} \tag{S28}$$

$$\tau_f = 1125 \cdot e^{-(V_m + 27)^2/240} + \frac{165}{1 + e^{(25 - V_m)/10}} + 80 \tag{S29}$$

$$\frac{df}{dt} = \frac{f_\infty - f}{\tau_f} \tag{S30}$$

L-type calcium current calcium-dependent inactivation gate

$$\alpha_{fCa} = \frac{1}{1 + [Ca_i/(3.25 \cdot 10^{-4})]^8} \tag{S31}$$

$$\beta_{fCa} = \frac{0.1}{1 + e^{(Ca_i - 0.0005)/1 \cdot 10^{-4}}} \tag{S32}$$

$$\gamma_{fCa} = \frac{0.2}{1 + e^{(Ca_i - 0.00075)/8 \cdot 10^{-4}}} \tag{S33}$$

$$fCa_\infty = \frac{\alpha_{fCa} + \beta_{fCa} + \gamma_{fCa} + 0.23}{1.46} \tag{S34}$$

$$k_{fCa} = \begin{cases} 0, & fCa_\infty < fCa \text{ and } V_m > -60 \text{ mV} \\ 1, & \text{otherwise} \end{cases} \tag{S35}$$

$$\frac{dfCa}{dt} = k_{fCa} \cdot \frac{fCa_\infty - fCa}{\tau_{fCa}} \tag{S36}$$

Transient Outward Potassium Current

$$i_{to} = g_{to} \cdot r \cdot s \cdot f_{Ca} \cdot (V_m - E_K) \tag{S37}$$

Transient outward potassium current activation gate

$$s_\infty = \frac{1}{1 + e^{(20 + V_m)/5}} \tag{S38}$$

$$\tau_s = 85 \cdot e^{-(V_m + 45)^2/320} + \frac{5}{1 + e^{(V_m - 20)/5}} + 3 \tag{S39}$$

$$\frac{ds}{dt} = \frac{s_\infty - s}{\tau_s} \tag{S40}$$

Transient outward potassium channel inactivation gate

$$r_\infty = \frac{1}{1 + e^{(20 - V_m)/6}} \tag{S41}$$

$$\tau_r = 9.5 \cdot e^{-(V_m + 40)^2/1800} + 0.8 \tag{S42}$$

$$\frac{dr}{dt} = \frac{r_\infty - r}{\tau_r} \tag{S43}$$

Slow Delayed Rectifier Potassium Current

$$i_{Ks} = g_{Ks} \cdot Xs^2 \cdot (V_m - E_{Ks}) \tag{S44}$$

Slow delayed rectifier potassium current activation gate

$$Xs_\infty = \frac{1}{1 + e^{(-5 - V_m)/14}} \tag{S45}$$

$$\alpha_{Xs} = \frac{1100}{\sqrt{1 + e^{(-10 - V_m)/6}}} \tag{S46}$$

$$\beta_{Xs} = \frac{0.1}{1 + e^{(V_m - 60)/20}} \quad (S47)$$

$$\tau_{Xs} = \alpha_{Xs} \cdot \beta_{Xs} \quad (S48)$$

$$\frac{dXs}{dt} = \frac{Xs_\infty - Xs}{\tau_{Xs}} \quad (S49)$$

Rapid Delayed Rectifier Potassium Current

$$i_{Kr} = g_{Kr} \cdot \sqrt{\frac{K_o}{5.4}} \cdot Xr1 \cdot Xr2 \cdot (V_m - E_K) \quad (S50)$$

Rapid delayed rectifier potassium current activation gate

$$Xr1_\infty = \frac{1}{1 + e^{(-26 - V_m)/7}} \quad (S51)$$

$$\alpha_{Xr1} = \frac{450}{1 + e^{(-45 - V_m)/10}} \quad (S52)$$

$$\beta_{Xr1} = \frac{6}{1 + e^{(V_m + 30)/11.5}} \quad (S53)$$

$$\tau_{Xr1} = \alpha_{Xr1} \cdot \beta_{Xr1} \quad (S54)$$

$$\frac{dXr1}{dt} = \frac{Xr1_\infty - Xr1}{\tau_{Xr1}} \quad (S55)$$

Rapid delayed rectifier potassium current inactivation gate

$$Xr2_\infty = \frac{1}{1 + e^{(88 + V_m)/24}} \quad (S56)$$

$$\alpha_{Xr2} = \frac{3}{1 + e^{(-60 - V_m)/20}} \quad (S57)$$

$$\beta_{Xr2} = \frac{1.12}{1 + e^{(V_m - 60)/20}} \quad (S58)$$

$$\tau_{Xr2} = \alpha_{Xr2} \cdot \beta_{Xr2} \quad (S59)$$

$$\frac{dXr2}{dt} = \frac{Xr2_\infty - Xr2}{\tau_{Xr2}} \quad (S60)$$

Inward Rectifier Potassium Current

$$i_{K1} = g_{K1} \cdot \sqrt{\frac{K_o}{5.4}} \cdot XK1_{\infty} \cdot (V_m - E_K) \tag{S61}$$

Inward rectifier potassium current activation gate

$$XK1_{\infty} = \frac{\alpha_{XK1}}{\alpha_{XK1} + \beta_{XK1}} \tag{S62}$$

$$\alpha_{XK1} = \frac{0.1}{1 + e^{0.06 \cdot (V_m - E_K - 200)}} \tag{S63}$$

$$\beta_{XK1} = 3 \cdot e^{0.0002 \cdot (V_m - E_K + 100)} \cdot \frac{e^{0.1 \cdot (V_m - E_K - 10)}}{1 + e^{-0.5 \cdot (V_m - E_K)}} \tag{S64}$$

Inward Rectifier Potassium Current

$$i_{NaCa} = K_{NaCa} \cdot \frac{\left(e^{\frac{\gamma_{NaCa} V_m F}{RT}} \cdot Na_i^3 \cdot Ca_o\right) - \left(e^{\frac{(\gamma_{NaCa} - 1) V_m F}{RT}} \cdot Na_o^3 \cdot Ca_i \cdot \alpha_{NaCa}\right)}{(K_{mNai}^3 + Na_o^3) \cdot (K_{mCa} + Ca_o) \cdot \left(1 + K_{sat} \cdot e^{\frac{(\gamma_{NaCa} - 1) V_m F}{RT}}\right)} \tag{S65}$$

Sodium Potassium Pump Current

$$i_{NaCa} = \frac{P_{NaK} K_o Na_i}{(K_o + K_{mK}) \cdot (Na_i + K_{mNa}) \cdot \left(1 + 0.1245 \cdot e^{\frac{-0.1 \cdot V_m F}{RT}} + 0.0353 \cdot e^{\frac{-V_m F}{RT}}\right)} \tag{S66}$$

Calcium Pump Current

$$i_{pCa} = g_{pCa} \cdot \frac{Ca_i}{K_{pCa} + Ca_i} \tag{S67}$$

Potassium Pump Current

$$i_{pK} = g_{pCa} \cdot \frac{V_m - E_K}{1 + e^{(25 - V_m)/5.98}} \tag{S68}$$

Sodium Background Current

$$i_{bNa} = g_{bNa} \cdot (V_m - E_{Na}) \tag{S69}$$

Calcium Background Current

$$i_{bCa} = g_{bCa} \cdot (V_m - E_{Ca}) \tag{S70}$$

Calcium Dynamics

$$i_{up} = \frac{Vmax_{up}}{1 + \left(K_{up}^2 / Ca_i^2\right)} \tag{S71}$$

$$i_{leak} = V_{leak} \cdot (Ca_{SR} - Ca_i) \tag{S72}$$

$$i_{rel} = \left(\frac{a_{rel}Ca_{SR}^2}{b_{rel}^2 + Ca_{SR}^2} + c_{rel}\right) \cdot d \cdot g \tag{S73}$$

SR calcium release gating

$$g_\infty = \begin{cases} {}^{1}\!/\![1 + (Ca_i^6/0.00035^6)] & Ca_i \leq 0.00035 \text{ mM} \\ {}^{1}\!/\![1 + (Ca_i^{16}/0.00035^{16})] & \text{otherwise} \end{cases} \tag{S74}$$

$$k_{gCa} = \begin{cases} 0, & g_\infty - g > 0 \text{ and } V_m > 60 \text{ mV} \\ 1, & \text{otherwise} \end{cases} \tag{S75}$$

$$\frac{dg}{dt} = k_{gCa} \cdot \frac{g_\infty - g}{\tau_g} \tag{S76}$$

SR and cytosolic calcium evolution with buffering

$$Cai_{bufc} = \frac{1}{1 + \left(Bufc \cdot K_{Bufc}\right)/\left(Ca_i + K_{Bufc}\right)^2} \tag{S77}$$

$$CaSR_{bufSR} = \frac{1}{1 + \left(BufSR \cdot K_{BufSR}\right)/\left(Ca_{SR} + K_{BufSR}\right)^2} \tag{S78}$$

$$\frac{dCa_i}{dt} = Cai_{bufc} \cdot \left\{i_{leak} - i_{up} + i_{rel} - C_m \cdot \left[\frac{i_{CaL} + i_{bCa} + i_{pCa} - (2 \cdot i_{NaCa})}{2 \cdot V_c F}\right]\right\} \tag{S79}$$

$$\frac{dCa_{SR}}{dt} = CaSR_{bufSR} \cdot \frac{V_c}{V_{SR}} \cdot \left(i_{up} - i_{rel} - i_{leak}\right) \tag{S80}$$

Sodium Dynamics

$$\frac{dNa_i}{dt} = -C_m \cdot \left[\frac{i_{Na} + i_{bNa} + (3 \cdot i_{NaK}) + (3 \cdot i_{NaCa})}{V_c F}\right] \tag{S81}$$

Potassium Dynamics

$$\frac{dK_i}{dt} = -C_m \cdot \left[\frac{i_{K1} + i_{to} + i_{Kr} + i_{Ks} + i_{pK} + i_{stim} - (2 \cdot i_{NaK})}{V_c F}\right] \tag{S82}$$

Model 2: Pandit et al. rat cardiomyocyte model

The set of equations given below represent the membrane ion channel, pumps and transporters along with sarcoplasmic reticulum function and calcium buffering for the model of rat cardiomyocyte electrophysiology and ion handling originally presented by Pandit et al.[2] A sodium dependent term has been added to the sodium-potassium pump current expression (S141) which was omitted in the original Pandit et al. manuscript.

Reversal Potentials

$$E_{Na} = \frac{RT}{F} \log \frac{Na_o}{Na_i} \tag{S83}$$

$$E_{Ca} = 65 \text{ mV} \tag{S84}$$

$$E_K = \frac{RT}{F} \log \frac{K_o}{K} \tag{S85}$$

Fast Sodium Current

$$I_{Na} = g_{Na} \cdot m^3 \cdot h \cdot j \cdot (V_m - E_{Na}) \tag{S86}$$

Fast sodium current activation gate

$$\bar{m} = \frac{1}{1 + e^{(V_m+45)/-6.5}} \tag{S87}$$

$$\tau_m = \frac{0.00136}{\frac{0.32 \cdot (V_m + 47.13)}{1 - e^{-0.1 \cdot (V_m + 47.13)}} + (0.08 \cdot e^{V_m/11})} \tag{S88}$$

$$\frac{dm}{dt} = \frac{\bar{m} - m}{\tau_m} \tag{S89}$$

Fast sodium current fast inactivation gate

$$\bar{h} = \frac{1}{1 + e^{(V_m-76.1)/6.07}} \tag{S90}$$

$$\tau_h = \begin{cases} 0.0004537 \cdot \left(1 + e^{-(V_m+10.66)/11.1}\right) & V_m \geq -40 \text{ mV} \\ \dfrac{0.00349}{0.135 \cdot e^{-(V_m+80)/6.8} + 3.56 \cdot e^{0.079 \cdot V_m} + 310000 \cdot e^{0.35 \cdot V_m}} & \text{otherwise} \end{cases} \tag{S91}$$

$$\frac{dh}{dt} = \frac{\bar{h} - h}{\tau_h} \tag{S92}$$

Fast sodium current slow inactivation gate

$$\bar{j} = \frac{1}{1 + e^{(V_m-76.1)/6.07}} \tag{S93}$$

$$\tau_j = \begin{cases} \dfrac{0.01163 \cdot 1 + e^{-0.1 \cdot (V_m+32)}}{e^{-2.535 \cdot 10^{-7} \cdot V_m}} & V_m \geq -40 \text{ mV} \\ \dfrac{0.00349}{\frac{(V_m + 37.78) \cdot (-127140 \cdot e^{0.2444 \cdot V_m} - 3.474 \cdot 10^{-5} \cdot e^{-0.04391 \cdot V_m})}{1 + e^{0.311 \cdot (V_m+79.23)}} + \frac{0.1212 \cdot e^{-0.01052 \cdot V_m}}{1 + e^{-0.1378 \cdot (V_m+40.14)}}} & \text{otherwise} \end{cases} \tag{S94}$$

$$\frac{dj}{dt} = \frac{\bar{j} - j}{\tau_j} \tag{S95}$$

L-Type Calcium Current

$$I_{CaL} = g_{CaL} \cdot d \cdot \left[\left(0.9 + \frac{Ca_{inact}}{10}\right) \cdot f_{11} + \left(0.1 - \frac{Ca_{inact}}{10}\right) \cdot f_{12}\right] \cdot (V_m - E_{Ca}) \tag{S96}$$

L-type calcium current activation gate

$$\bar{d} = \frac{1}{1 + e^{(V_m+15.3)/-5}} \tag{S97}$$

$$\tau_d = 0.00305 \cdot e^{-0.0045\cdot(V_m+7)^2} + 0.00105 \cdot e^{-0.002\cdot(V_m-18)^2} + 0.00025 \tag{S98}$$

$$\frac{dd}{dt} = \frac{\bar{d} - d}{\tau_d} \tag{S99}$$

L-type calcium current fast inactivation gate

$$\bar{f}_{11} = \frac{1}{1 + e^{(V_m+26.7)/5.4}} \tag{S100}$$

$$\tau_{f11} = 0.105 \cdot e^{-[(V_m+45)/12]^2} + \frac{0.04}{1 + e^{(-V_m+25)/25}} + \frac{0.015}{1 + e^{(V_m+75)/25}} + 0.0017 \tag{S101}$$

$$\frac{df_{11}}{dt} = \frac{\bar{f}_{11} - f_{11}}{\tau_{f11}} \tag{S102}$$

L-type calcium current slow inactivation gate

$$\bar{f}_{12} = \frac{1}{1 + e^{(V_m+26.7)/5.4}} \tag{S103}$$

$$\tau_{f12} = 0.041 \cdot e^{-[(V_m+47)/12]^2} + \frac{0.08}{1 + e^{(V_m+55)/-5}} + \frac{0.015}{1 + e^{(V_m+75)/25}} + 0.0017 \tag{S104}$$

$$\frac{df_{12}}{dt} = \frac{\bar{f}_{12} - f_{12}}{\tau_{f12}} \tag{S105}$$

L-type calcium current calcium-dependent inactivation gate

$$\overline{Ca}_{1inact} = \frac{1}{1 + Ca_{ss}/0.01} \tag{S106}$$

$$\tau_{Cainact} = 0.009 \tag{S107}$$

$$\frac{dCa_{inact}}{dt} = \frac{\overline{Ca}_{inact} - Ca_{inact}}{\tau_{Cainact}} \tag{S108}$$

Calcium-Independent Transient Outward Potassium Current

$$I_t = g_t \cdot r \cdot [(a \cdot s) + (b \cdot s_{slow})] \cdot (V_m - E_K) \tag{S109}$$

$$a = 0.886 \tag{S110}$$

$$b = 0.114 \tag{S111}$$

Calcium-independent transient outward potassium current activation gate

$$\bar{r} = \frac{1}{1 + e^{(V_m + 10.6)/-11.42}} \tag{S112}$$

$$\tau_r = \frac{1}{45.16 \cdot e^{0.03577 \cdot (V_m + 50)} + 98.9 \cdot e^{-0.1 \cdot (V_m + 38)}} \tag{S113}$$

$$\frac{dr}{dt} = \frac{\bar{r} - r}{\tau_r} \tag{S114}$$

Calcium-independent transient outward potassium current fast inactivation gate

$$\bar{s} = \frac{1}{1 + e^{(V_m + 45.3)/6.8841}} \tag{S115}$$

$$\tau_s = 0.35 \cdot e^{-[(V_m + 70)/15]^2} + 0.035 \tag{S116}$$

$$\frac{ds}{dt} = \frac{\bar{s} - s}{\tau_s} \tag{S117}$$

Calcium-independent transient outward potassium current slow inactivation gate

$$\bar{s}_{slow} = \frac{1}{1 + e^{(V_m + 45.3)/6.8841}} \tag{S118}$$

$$\tau_{sslow} = 3.7 \cdot e^{-[(V_m + 70)/30]^2} + 0.035 \tag{S119}$$

$$\frac{ds_{slow}}{dt} = \frac{\bar{s}_{slow} - s_{slow}}{\tau_{sslow}} \tag{S120}$$

Steady-State Outward Potassium Current

$$I_{ss} = g_{ss} \cdot r_{ss} \cdot s_{ss} \cdot (V_m - E_K) \tag{S121}$$

Steady-state outward potassium current activation gate

$$\bar{r}_{ss} = \frac{1}{1 + e^{(V_m+11.5)/-11.82}} \tag{S122}$$

$$\tau_{rss} = \frac{10}{45.16 \cdot e^{0.03577 \cdot (V_m+50)} + 98.9 \cdot e^{-0.1 \cdot (V_m+38)}} \tag{S123}$$

$$\frac{dr_{ss}}{dt} = \frac{\bar{r}_{ss} - r_{ss}}{\tau_{rss}} \tag{S124}$$

Steady-state outward potassium current inactivation gate

$$\bar{s}_{ss} = \frac{1}{1 + e^{(V_m+87.5)/10.3}} \tag{S125}$$

$$\tau_{sss} = 2.1 \text{ ms} \tag{S126}$$

$$\frac{ds_{ss}}{dt} = \frac{\bar{s}_{ss} - s_{ss}}{\tau_{sss}} \tag{S127}$$

Inward Rectifier Potassium Current

$$I_{K1} = \left(\frac{48}{e^{\frac{V_m+37}{25}} + e^{\frac{V_m+37}{-25}}} + 10 \right) \cdot \left(\frac{0.001}{1 + e^{\frac{V_m - E_K - 76.66}{-17}}} \right) \cdot \left\{ \frac{g_{K1} \cdot (V_m - E_{K1} - 1.73)}{\left[1 + e^{\frac{1.613 \cdot F \cdot (V_m - E_{K1} - 1.73)}{RT}} \right] \cdot \left(1 + e^{\frac{K_o - 0.9988}{-0.124}} \right)} \right\} \tag{S128}$$

Hyperpolarization-Activated Cation Current

$$I_f = I_{fNa} + I_{fK} \tag{S129}$$

$$I_{fNa} = g_f \cdot y \cdot f_{Na} \cdot (V_m - E_{Na}) \tag{S130}$$

$$I_{fK} = g_f \cdot y \cdot f_K \cdot (V_m - E_K) \tag{S131}$$

$$f_{Na} = 0.2 \tag{S132}$$

$$f_K = 1 - f_{Na} \tag{S133}$$

Hyperpolarization-activated cation current inactivation gate

$$y_\infty = \frac{1}{1 + e^{(V_m+138.6)/10.48}} \tag{S134}$$

$$\tau_y = \frac{1}{0.11885 \cdot e^{(V_m+80)/28.37} + 0.5623 \cdot e^{(V_m+80)/-14.19}} \tag{S135}$$

$$\frac{dy}{dt} = \frac{y_\infty - y}{\tau_y} \tag{S136}$$

Sodium, Potassium, Calcium and Total Background Currents

$$I_{BNa} = g_{BNa} \cdot (V_m - E_{Na}) \tag{S137}$$

$$I_{BK} = g_{BK} \cdot (V_m - E_K) \tag{S138}$$

$$I_{BCa} = g_{BCa} \cdot (V_m - E_{Ca}) \tag{S139}$$

$$I_B = I_{BNa} + I_{BK} + I_{BCa} \tag{S140}$$

Sodium-Potassium Pump Current

$$I_{NaK} = \bar{I}_{NaK} \cdot \frac{1}{1 + 0.1245 \cdot e^{-0.1 \cdot V_m F/RT} + 0.0365 \cdot \sigma \cdot e^{-V_m F/RT}} \cdot \frac{K_o}{K_o + k_{mK}} \cdot \frac{1}{1 + (k_{mNa}/Na_i)^{1.5}} \tag{S141}$$

$$\sigma = \frac{e^{Na_o/67.3} - 1}{7} \tag{S142}$$

Sarcolemmal Calcium Pump Current

$$I_{CaP} = \bar{I}_{CaP} \cdot \frac{Ca_i}{Ca_i + 0.0004} \cdot \tag{S143}$$

Sodium-Calcium Exchanger Current

$$I_{NaCa} = k_{NaCa} \cdot \frac{Na_i^3 Ca_o \cdot e^{0.03743 \cdot \gamma_{NaCa} \cdot V_m} - Na_o^3 Ca_i \cdot e^{0.03743 \cdot (\gamma_{NaCa} - 1) \cdot V_m}}{1 + d_{NaCa} \cdot (Na_o^3 Ca_i + Na_i^3 Ca_o)} \tag{S144}$$

Calcium Release from Sarcoplasmic Reticulum

$$J_{rel} = v_1 \cdot (P_{o1} + P_{o2}) \cdot (Ca_{JSR} - Ca_{ss}) \tag{S145}$$

Ryanodine receptor four state model

$$\frac{dP_{o1}}{dt} = \left(k_{ap} \cdot Ca_{ss}^{n_{RyR}} \cdot P_{c1}\right) - (k_{am} \cdot P_{o1}) - \left(k_{bp} \cdot Ca_{ss}^{m_{RyR}} \cdot P_{o1}\right) + (k_{bm} \cdot P_{o2}) - \left(k_{cp} \cdot P_{o1}\right) + (k_{cm} \cdot P_{c2}) \tag{S146}$$

$$\frac{dP_{o2}}{dt} = \left(k_{bp} \cdot Ca_{ss}^{m_{RyR}} \cdot P_{o1}\right) - (k_{bm} \cdot P_{o2}) \tag{S147}$$

$$\frac{dP_{c1}}{dt} = \left(-k_{ap} \cdot Ca_{ss}^{n_{RyR}} \cdot P_{c1}\right) + (k_{am} \cdot P_{o1}) \tag{S148}$$

$$\frac{dP_{c2}}{dt} = \left(k_{cp} \cdot P_{o1}\right) + \left(k_{cm} \cdot P_{c2}\right) \tag{S149}$$

Calcium Uptake by Sarcoplasmic Reticulum SERCA2a

$$J_{up} = K_{SR} \cdot \frac{\left(v_{maxf} \cdot f_b\right) - \left(v_{maxr} \cdot r_b\right)}{1 + f_b + r_b} \tag{S150}$$

$$f_b = \left(\frac{Ca_i}{K_{fb}}\right)^{N_{fb}} \tag{S151}$$

$$r_b = \left(\frac{Ca_i}{K_{rb}}\right)^{N_{rb}} \tag{S152}$$

Calcium Transport and Buffering in Cytosol, Subspace and Sarcoplasmic Reticulum

$$J_{tr} = \frac{Ca_{NSR} - Ca_{JSR}}{\tau_{tr}} \tag{S153}$$

$$J_{xfer} = \frac{Ca_{ss} - Ca_i}{\tau_{xfer}} \tag{S154}$$

$$J_{trpn} = \frac{dHTRPN_{Ca}}{dt} + \frac{dLTRPN_{Ca}}{dt} \tag{S155}$$

$$\frac{dHTRPN_{Ca}}{dt} = \left[k_{htrpnp} \cdot Ca_i \cdot \left(HTRPN_{tot} - HTRPN_{Ca}\right)\right] - \left(k_{htrpnm} \cdot HTRPN_{Ca}\right) \tag{S156}$$

$$\frac{dLTRPN_{Ca}}{dt} = \left[k_{ltrpnp} \cdot Ca_i \cdot \left(LTRPN_{tot} - LTRPN_{Ca}\right)\right] - \left(k_{ltrpnm} \cdot LTRPN_{Ca}\right) \tag{S157}$$

Sodium, Potassium and Calcium Handling in Cytosol

$$\frac{dNa_i}{dt} = \frac{-\left[I_{Na} + I_{BNa} + \left(3 \cdot I_{NaCa}\right) + \left(3 \cdot I_{NaK}\right) + I_{fNa}\right]}{V_{myo}F} \tag{S158}$$

$$\frac{dK_i}{dt} = \frac{-\left[I_{ss} + I_{BK} + I_t + I_{K1} + I_{fK} + \left(2 \cdot I_{NaK}\right)\right]}{V_{myo}F} \tag{S159}$$

$$\frac{dCa_i}{dt} = \beta_i \cdot \left\{\left(J_{xfer} - J_{up} - J_{trpn}\right) - \frac{\left[I_{BCa} - \left(2 \cdot I_{NaCa}\right) + I_{CaP}\right]}{2 \cdot V_{myo}F}\right\} \tag{S160}$$

$$\beta_i = \frac{1}{1 + \frac{CMDN_{tot} \cdot K_{mCMDN}}{\left(K_{mCMDN} + Ca_i\right)^2} + \frac{EGTA_{tot} \cdot K_{mEGTA}}{\left(K_{mEGTA} + Ca_i\right)^2}} \tag{S161}$$

Calcium Handling in Subspace and Sarcoplasmic Reticulum

$$\frac{dCa_{ss}}{dt} = \beta_{ss} \cdot \left[\left(\frac{J_{rel} \cdot V_{JSR}}{V_{ss}}\right) - \left(\frac{J_{xfer} \cdot V_{myo}}{V_{ss}}\right) - \left(\frac{I_{CaL}}{2 \cdot V_{ss} F}\right)\right] \quad \text{(S162)}$$

$$\frac{dCa_{JSR}}{dt} = \beta_{JSR} \cdot (J_{tr} - J_{rel}) \quad \text{(S163)}$$

$$\frac{dCa_{NSR}}{dt} = \left(\frac{J_{up} \cdot V_{myo}}{V_{NSR}}\right) - \left(\frac{J_{tr} \cdot V_{JSR}}{V_{NSR}}\right) \quad \text{(S164)}$$

$$\beta_{ss} = \frac{1}{1 + \frac{CMDN_{tot} \cdot K_{mCMDN}}{(K_{mCMDN} + Ca_{ss})^2}} \quad \text{(S165)}$$

$$\beta_{JSR} = \frac{1}{1 + \frac{CSQN_{tot} \cdot K_{mCSQN}}{\left(K_{mCSQN} + Ca_{JSR}\right)^2}} \quad \text{(S166)}$$

Model 3: MacCannell et al. fibroblast model

The set of equations given below represent the membrane ion channels and sodium/potassium pump for the model of the generic mammalian fibroblast originally presented by MacCannell et al.[3] Since the original MFB model uses the units of time of milliseconds, equations for the gating time constants on the voltage-dependent potassium channel are different from the original model when coupled to the PCM which has a base unit of seconds as indicated in equations S170 and S173. None of the fixed parameters used in equations S167 - S179 (g_{Kv}, g_{K1}, G_{Nab}, $\bar{I}_{NaK}$, K_{mK}, K_{mNa}, V_{rev}, B and E_K) are in explicit units of milliseconds so no correction is needed in the equations or in the fixed parameters as seen in Section 2 when coupled with the different CM models.

Reversal Potential

$$E_{Na} = \frac{RT}{F} \cdot \log \frac{Na_o}{Na_i} \quad \text{(S167)}$$

Voltage-Dependent Potassium Current

$$I_{Kv} = g_{Kv} \cdot r_{Kv} \cdot s_{Kv} \cdot (V_m - E_K) \quad \text{(S168)}$$

Voltage-dependent potassium current activation gate

$$\bar{r}_{Kv} = \frac{1}{1 + e^{-(V_m+20)/11}} \quad \text{(S169)}$$

$$\tau_{rKv} = \begin{cases} 20.3 + 138 \cdot e^{-[(V_m+20)/25.9]^2} & \text{when coupled with tTCM} \\ 0.0203 + 0.138 \cdot e^{-[(V_m+20)/25.9]^2} & \text{when coupled with PCM} \end{cases} \quad \text{(S170)}$$

$$\frac{dr_{Kv}}{dt} = \frac{\bar{r}_{Kv} - r_{Kv}}{\tau_{rKv}} \tag{S171}$$

Voltage-dependent potassium current inactivation gate

$$\bar{s}_{Kv} = \frac{1}{1 + e^{-(V_m+23)/7}} \tag{S172}$$

$$\tau_{sKv} = \begin{cases} 1574 + 5268 \cdot e^{-[(V_m+23)/22.7]^2} & \text{when coupled with tTCM} \\ 1.574 + 5.268 \cdot e^{-[(V_m+23)/22.7]^2} & \text{when coupled with PCM} \end{cases} \tag{S173}$$

$$\frac{ds_{Kv}}{dt} = \frac{\bar{s}_{Kv} - s_{Kv}}{\tau_{sKv}} \tag{S174}$$

<u>*Inward Rectifier Potassium Current*</u>

$$I_{K1} = g_{K1} \cdot \frac{\alpha_{K1} \cdot (V_m - E_K)}{\alpha_{K1} + \beta_{K1}} \tag{S175}$$

$$\alpha_{K1} = \frac{0.1}{1 + e^{0.06 \cdot (V_m - E_K - 200)}} \tag{S176}$$

$$\beta_{K1} = \frac{3 \cdot e^{0.0002 \cdot (V_m - E_K + 100)} + e^{0.1 \cdot (V_m - E_K - 10)}}{1 + e^{-0.5 \cdot (V_m - E_K)}} \tag{S177}$$

<u>*Sodium/Potassium Pump Current*</u>

$$I_{NaK} = \bar{I}_{NaK} \cdot \frac{K_o}{K_o + K_{mK}} \cdot \frac{Na_i^{1.5}}{Na_i^{1.5} + K_{mNa}^{1.5}} \cdot \frac{V_m - V_{rev}}{V_m - B} \tag{S178}$$

<u>*Background Sodium Current*</u>

$$I_{bNa} = G_{Nab} \cdot (V_m - E_{Na}) \tag{S179}$$

Model 4: Sachse et al. fibroblast model

The equations presented below delineate the membrane ion channels pertinent to the model of a generic mammalian fibroblast, as initially described by Sachse et al. In the SFB model, the cytosolic potassium concentration (K_i) is maintained at a constant value of 140 mM. However, in our approach, we have opted to align K_i with the cytosolic potassium concentration observed in the coupled cardiomyocyte model, which is permitted to vary. This concentration is subsequently utilized to compute the Nernst potential (E_K) as articulated in equation S180. The leading constant in equation S183, when integrated with the tTCM, serves to convert the differing units of Faraday's constant (F) from C/mmole to C/mol, as well as the time units from milliseconds to seconds, thereby facilitating the expression of current in nanoamperes (nA). Furthermore, all rate constants (k_v, k_{mv}, k_o, k_{mo}) that dictate the kinetics of the four-state Shaker potassium channel, along with the ion channel permeability (P_{Shkr}), are adjusted by a factor of 1000 to convert their units from per second to per millisecond in the coupled tTCM/SFB model, as detailed in Section 2.

Reversal Potential

$$E_K = \frac{RT}{F} \cdot \log \frac{K_o}{K_i} \tag{S180}$$

Inward Rectifier Potassium Current

$$I_{Kir} = G_{Kir} \cdot O_{Kir} \cdot \sqrt{K_o \cdot 0.001} \cdot (V_m - E_K) \tag{S181}$$

$$O_{Kir} = \frac{1}{a_{Kir} + e^{[b_{Kir} \cdot (V_m - E_K) \cdot F]/RT}} \tag{S182}$$

Shaker Potassium Current

$$I_{Shkr} = \begin{cases} 10^6 \cdot P_{Shkr} \cdot O_{Shkr} \cdot \frac{V_m F^2}{RT} \cdot \frac{K_i - K_o \cdot e^{-V_m F/RT}}{1 - e^{-V_m F/RT}} & \text{when coupled with tTCM} \\ P_{Shkr} \cdot O_{Shkr} \cdot \frac{V_m F^2}{RT} \cdot \frac{K_i - K_o \cdot e^{-V_m F/RT}}{1 - e^{-V_m F/RT}} & \text{when coupled with PCM} \end{cases} \tag{S183}$$

Shaker channel open probability six state model

$$k_v = k_{v0} \cdot e^{V_m z_v F/RT} \tag{S184}$$

$$k_{mv} = k_{mv0} \cdot e^{V_m z_{mv} F/RT} \tag{S185}$$

$$\frac{dC0_{Shkr}}{dt} = (-4k_v \cdot C0_{Shkr}) + (k_{mv} \cdot C1_{Shkr}) \tag{S186}$$

$$\frac{dC1_{Shkr}}{dt} = (4k_v \cdot C0_{Shkr}) - [(3k_v + k_{mv}) \cdot C1_{Shkr}] + (2k_{mv} \cdot C2_{Shkr}) \tag{S187}$$

$$\frac{dC2_{Shkr}}{dt} = (3k_v \cdot C1_{Shkr}) - [(2k_v + 2k_{mv}) \cdot C2_{Shkr}] + (3k_{mv} \cdot C3_{Shkr}) \tag{S188}$$

$$\frac{dC3_{Shkr}}{dt} = (2k_v \cdot C2_{Shkr}) - [(k_v + 3k_{mv}) \cdot C3_{Shkr}] + (4k_{mv} \cdot C4_{Shkr}) \tag{S189}$$

$$\frac{dC4_{Shkr}}{dt} = (k_v \cdot C3_{Shkr}) - [(k_o + 4k_{mv}) \cdot C4_{Shkr}] + (k_{mo} \cdot O_{Shkr}) \tag{S190}$$

$$\frac{dO_{Shkr}}{dt} = (k_o \cdot C4_{Shkr}) - (k_{mo} \cdot O_{Shkr}) \tag{S191}$$

Nonselective Background Current

$$I_b = G_b \cdot (V_m - E_b) \tag{S192}$$

Shared Coupled Model Equations

These equations are used to calculate the current between the CM and FB along with the changes in membrane potential for each of the cell types. The expression for the CM/FB current is common to all four coupling combinations and results in units of nanoamperes. The equations for the updates of V_m in both the CM and FB however are different for the different combinations as a result of the differences in original units of the four models.

Cardiomyocyte-Fibroblast Current

$$I_{CMFB} = \frac{(V_{m,CM} - V_{m,FB})}{R_{Gap}} \tag{S193}$$

Cardiomyocyte and Fibroblast Membrane Potential: tTCM/MFB Coupled Model

$$\frac{dV_{m,tTCM}}{dt} = -1 \cdot \left(i_{ion,tTCM} + \frac{N_{FB} \cdot I_{CMFB}}{C_{m,tTCM} \cdot 1000} + I_{Stim} \right) \tag{S194}$$

$$\begin{aligned} i_{ion,tTCM} = {} & i_{Na,tTCM} + i_{CaL,tTCM} + i_{to,tTCM} + i_{Ks,tTCM} + i_{Kr,tTCM} + i_{K1,tTCM} + i_{NaCa,tTCM} + i_{NaK,tTCM} + i_{pCa,tTCM} \\ & + i_{pK,tTCM} + i_{bNa,tTCM} + i_{bCa,tTCM} \end{aligned} \tag{S195}$$

$$\frac{dV_{m,MFB}}{dt} = -1 \cdot \left(I_{Kv,MFB} + I_{K1,MFB} + I_{NaK,MFB} + I_{bNa,MFB} - \frac{I_{CMFB}}{C_{m,MFB}/1000} \right) \tag{S196}$$

Cardiomyocyte and Fibroblast Membrane Potential: tTCM/SFB Coupled Model

$$\frac{dV_{m,tTCM}}{dt} = -1 \cdot \left(i_{ion,tTCM} + \frac{N_{FB} \cdot I_{CMFB}}{C_{m,tTCM} \cdot 1000} + I_{Stim} \right) \tag{S194}$$

$$\begin{aligned} i_{ion,tTCM} = {} & i_{Na,tTCM} + i_{CaL,tTCM} + i_{to,tTCM} + i_{Ks,tTCM} + i_{Kr,tTCM} + i_{K1,tTCM} + i_{NaCa,tTCM} + i_{NaK,tTCM} + i_{pCa,tTCM} \\ & + i_{pK,tTCM} + i_{bNa,tTCM} + i_{bCa,tTCM} \end{aligned} \tag{S195}$$

$$\frac{dV_{m,SFB}}{dt} = -1 \cdot \frac{I_{Kir,SFB} + I_{Shkr,SFB} + I_{b,SFB} - I_{CMFB}}{C_{m,SFB} \cdot 1000} \tag{S196}$$

Cardiomyocyte and Fibroblast Membrane Potential: PCM/MFB Coupled Model

$$\frac{dV_{m,PCM}}{dt} = -1 \cdot \left(\frac{I_{ion,PCM} + (N_{FB} \cdot I_{CMFB}) + I_{Stim}}{C_{m,PCM}} \right) \tag{S197}$$

$$I_{ion,PCM} = I_{Na,PCM} + I_{CaL,PCM} + I_{t,PCM} + I_{ss,PCM} + I_{f,PCM} + I_{K1,PCM} + I_{B,PCM} + I_{NaK,PCM} + I_{NaCa,PCM} + I_{CaP,PCM} \tag{S198}$$

$$\frac{dV_{m,MFB}}{dt} = -1 \cdot \left[1000 \cdot \left(I_{Kv,MFB} + I_{K1,MFB} + I_{NaK,MFB} + I_{bNa,MFB} - I_{CMFB}\right) - \left(\frac{I_{CMFB}}{C_{m,MFB}} \cdot 10^{6}\right)\right] \tag{S199}$$

Cardiomyocyte and Fibroblast Membrane Potential: PCM/SFB Coupled Model

$$\frac{dV_{m,PCM}}{dt} = -1 \cdot \left(\frac{I_{ion,PCM} + (N_{FB} \cdot I_{CMFB}) + I_{Stim}}{C_{m,PCM}}\right) \tag{S200}$$

$$I_{ion,PCM} = I_{Na,PCM} + I_{CaL,PCM} + I_{t,PCM} + I_{ss,PCM} + I_{f,PCM} + I_{K1,PCM} + I_{B,PCM} + I_{NaK,PCM} + I_{NaCa,PCM} + I_{CaP,PCM} \tag{S201}$$

$$\frac{dV_{m,SFB}}{dt} = -1 \cdot \frac{I_{Kir,SFB} + I_{Shkr,SFB} + I_{b,SFB} - I_{CMFB}}{C_{m,SFB}} \tag{S202}$$

2. SUPPLEMENTARY FIGURE

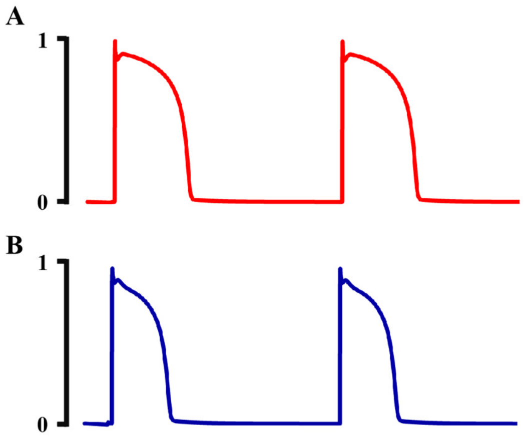

Figure S1. Fluorescence recordings of normalized action potential traces, comparing conditions with the absence of fibroblasts (A) to those with their presence (B).

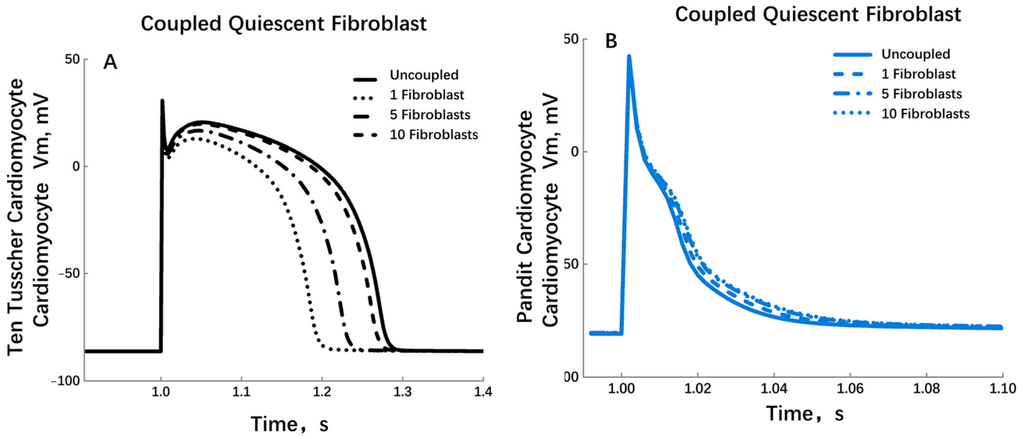

Figure S2. Cardiomyocyte action potential profiles when the quiescent fibroblasts model are coupled in turn with the cardiomyocyte models of ten Tusscher et al. and Pandit et al. The tTCM action potentials are shown when coupled with increasing numbers of fibroblasts in S(A). The PCM action potential when coupled similarly is shown in S(B). Gap junction resistance between cardiomyocyte and fibroblast is held constant in all 2 simulations at 100 MΩ.

Note: the quiescent fibroblast model, which was referred to in previous research[4], is described by the following equation:

$$I_f = G_f(V_f - E_f) \qquad \text{(S203)}$$

Where $E_f = -85\, mV,\ G_f = 0.103 nS/pF$

References

1 ten Tusscher, K. H. W. J., Noble, D., Noble, P. J. & Panfilov, A. V. A model for human ventricular tissue. *American Journal of Physiology-Heart and Circulatory Physiology* **286**, H1573-H1589 (2004).

2 Pandit, S. V., Clark, R. B., Giles, W. R. & Demir, S. S. A mathematical model of action potential heterogeneity in adult rat left ventricular myocytes. *Biophysical Journal* **81**, 3029-3051 (2001).

3 MacCannell, K. A. *et al.* A mathematical model of electrotonic interactions between ventricular myocytes and fibroblasts. *Biophysical Journal* **92**, 4121-4132, doi:Doi 10.1529/Biophysj.106.101410 (2007).

4 Xie, Y. *et al.* Effects of fibroblast-myocyte coupling on cardiac conduction and vulnerability to reentry: A computational study. *Heart Rhythm* **6**, 1641-1649, doi:http://dx.doi.org/10.1016/j.hrthm.2009.08.003 (2009).
